# The Roles of White Adipose Tissue and Liver NADPH in Dietary Restriction-Induced Longevity

**DOI:** 10.3390/antiox13070820

**Published:** 2024-07-08

**Authors:** Leah E. Jamerson, Patrick C. Bradshaw

**Affiliations:** Department of Biomedical Sciences, James H. Quillen College of Medicine, East Tennessee State University, Johnson City, TN 37614, USA

**Keywords:** aging, dietary restriction, NADPH, lifespan, metabolism, white adipose

## Abstract

Dietary restriction (DR) protocols frequently employ intermittent fasting. Following a period of fasting, meal consumption increases lipogenic gene expression, including that of NADPH-generating enzymes that fuel lipogenesis in white adipose tissue (WAT) through the induction of transcriptional regulators SREBP-1c and CHREBP. SREBP-1c knockout mice, unlike controls, did not show an extended lifespan on the DR diet. WAT cytoplasmic NADPH is generated by both malic enzyme 1 (ME1) and the pentose phosphate pathway (PPP), while liver cytoplasmic NADPH is primarily synthesized by folate cycle enzymes provided one-carbon units through serine catabolism. During the daily fasting period of the DR diet, fatty acids are released from WAT and are transported to peripheral tissues, where they are used for beta-oxidation and for phospholipid and lipid droplet synthesis, where monounsaturated fatty acids (MUFAs) may activate Nrf1 and inhibit ferroptosis to promote longevity. Decreased WAT NADPH from PPP gene knockout stimulated the browning of WAT and protected from a high-fat diet, while high levels of NADPH-generating enzymes in WAT and macrophages are linked to obesity. But oscillations in WAT [NADPH]/[NADP^+^] from feeding and fasting cycles may play an important role in maintaining metabolic plasticity to drive longevity. Studies measuring the WAT malate/pyruvate as a proxy for the cytoplasmic [NADPH]/[NADP^+^], as well as studies using fluorescent biosensors expressed in the WAT of animal models to monitor the changes in cytoplasmic [NADPH]/[NADP^+^], are needed during ad libitum and DR diets to determine the changes that are associated with longevity.

## 1. Introduction

Oxidation–reduction (redox) reactions play a fundamental role in regulating cellular metabolism. In humans, 426 different enzymes are regulated by the [NAD^+^]/[NADH] redox couple, the [NADPH]/[NADP^+^] redox couple, or one of the nucleotides from these couples [1]. Of the enzymes regulated by these redox couples, it is estimated that roughly 75% of them are regulated by [NAD^+^]/[NADH] and roughly 25% are regulated by [NADPH]/[NADP^+^], although there is some overlap, as some enzymes can bind both NAD(H) and NADP(H) (i.e., the mitochondrial enzymes glutamate dehydrogenase (GLUD1) [2], nicotinamide nucleotide transhydrogenase (NNT), and the folate cycle enzyme methylene tetrahydrofolate dehydrogenase 2 (MTHFD2) [3]). Overall, 352 of these 426 genes are expressed in the liver, and 30% of these hepatic genes showed opposing changes in gene expression during fasting and refeeding [1].

Almost all organisms show an oxidizing redox imbalance with aging [4], with only certain tissues being affected [5] and with mitochondria being more profoundly affected than other cellular compartments [6]. Oxidizing redox imbalance is primarily driven by mitochondrial-derived superoxide that is dismutated to hydrogen peroxide (H_2_O_2_), leading to the oxidation of macromolecules, including the formation of protein disulfides and glutathione disulfide from free sulfhydryls, unless the H_2_O_2_ is first detoxified by glutathione peroxidases, peroxiredoxins, or catalase. NADPH is oxidized by the enzymes glutathione disulfide reductase (GSR) and thioredoxin reductase that function in the restoration of sulfhydryl formation, resulting in an oxidized (decreased) [NADPH]/[NADP^+^] in some tissues with aging. The increased reactive oxygen species (ROS) generation with aging induces a compensatory increase in antioxidant gene expression. For example, aging caused the increased expression of mitochondrial superoxide dismutase 2 (SOD2), peroxiredoxin 5 (PRDX5), and uncoupling protein 2 (UCP2) in several aged rat tissues, and the increased expression of these genes with aging was blunted by dietary restriction (DR) [7], preventing the aging-induced increase in mitochondrial superoxide generation [8]. However, at the protein level in white adipose tissue (WAT), one antioxidant enzyme, peroxiredoxin 3 (PRDX3), showed the opposite pattern, showing decreased abundance with age and the decrease was prevented by DR, while WAT glutathione peroxidase 1 (GPX1) abundance was also increased by DR [9].

## 2. WAT and Liver Are the Primary Lipogenic Tissues and Therefore, Have a High Capacity for Cytoplasmic NADPH Generation for Lipid Synthesis

Following a carbohydrate-rich meal, glucose is taken up from the bloodstream by tissues to fuel energy metabolism, while the excess glucose in the bloodstream is primarily taken up by the lipogenic tissues of the WAT and liver, where much of the glucose can be used for fatty acid and triglyceride synthesis, while the liver (and skeletal muscles) also stores some of the glucose as glycogen. To synthesize one 16-carbon fatty acid (palmitate) from glucose-derived acetyl-CoA, it requires the oxidation of 14 molecules of NADPH. To synthesize one 27-carbon molecule of cholesterol, between 16 and 21 molecules of NADPH must be oxidized [10]. There is a range in the number of NADPH molecules oxidized in part due to the fact that the ER cholesterol synthesis enzyme NSDHL can use either NADH or NADPH as a coenzyme [11]. NADPH metabolism has been extensively studied in relation to the liver while being highly understudied in WAT. In the last decade, several studies have linked WAT lipogenesis with longevity [12], while no such link was found between hepatic lipogenesis and longevity.

The strongest links between DR-mediated longevity and hepatic NADPH appear to be the increased hepatic cytoplasmic [NADPH]/[NADP^+^] that occurs during DR [5] and the increased hepatic expression of NADPH-dependent flavin monooxygenase 3 (FMO3) during DR. The increased expression of hepatic FMO3 also occurs in several other long-lived mouse models [13,14]. In addition, *C. elegans* flavin monooxygenase homologs FMO-2 and FMO-4, which are predicted to be ER transmembrane proteins, are required for the extended lifespan of several longevity paradigms, including DR [15,16]. FMO-2 was identified as extending longevity by modulating one-carbon metabolism and methylation reactions, possibly by metabolizing tryptophan to N-formylkynurenine, which can then be metabolized to L-kynurenine releasing formic acid to increase folate cycle flux [15]. FMO-4 was shown to function downstream of FMO-2 in the longevity pathway and to genetically interact with proteins involved in ER-mitochondrial Ca^2+^ flux. Altered Ca^2+^ signaling led to increased FMO-4 expression through decreased transcriptional repression of FMO-4 by the ER stress response transcriptional regulator ATF-6 [16]. In this review, since knowledge of WAT NADPH metabolism is frequently lacking, we will describe what is known about hepatic NADPH metabolism during aging and DR. In many conditions, with the major exceptions of the increased rate of WAT lipogenesis following meals during the DR diet [17] and the primary enzymes responsible for cytoplasmic NADPH synthesis [18,19], liver lipogenic metabolism shares many similarities to that of WAT.

## 3. No Change in the Liver Cytoplasmic [NAD^+^]/[NADH] or [NADPH]/[NADP^+^] in Aged Mice

With aging, there is a loss of cellular NAD^+^ in some tissues due to increased NAD^+^ degradation [20] as well as a reduction in [NAD^+^]/[NADH] [21]. A study using C57BL/6J mice found no significant changes in the liver cytoplasmic [NAD^+^]/[NADH] or [NADPH]/[NADP^+^] between 3 and 30 months of age, as measured in relation to pyruvate/lactate and malate/pyruvate ratios, respectively [5]. What is unique about the liver that protects it from cytoplasmic redox changes with aging? The liver possesses moderately high levels of cytoplasmic malic enzyme 1 (ME1), which reduces NADP^+^ to NADPH, and high levels of cytoplasmic lactate dehydrogenase (LDH) that oxidizes NADH to NAD^+^. When ME1 levels are high, the malate/pyruvate ratio strongly determines cytoplasmic [NADPH]/[NADP^+^]. Likewise, when LDH levels are high, the pyruvate/lactate ratio strongly determines the cytoplasmic [NAD^+^]/[NADH] [22]. The presence of pyruvate in the numerator of one of these ratios and the denominator of the other links these ratios together, driving both sets of redox couples to become oxidized or reduced together or not at all. This relationship was first discovered by Hans Krebs’ lab studying liver [23] and later confirmed by Barbara Corkey’s lab [24]. The linking of the [NAD^+^]/[NADH] and [NADPH]/[NADP^+^] ratios in the liver may partly explain their lack of change with aging. In other tissues with low ME1 levels, aging may cause [NAD^+^]/[NADH] to become reduced, while the [NADPH]/[NADP^+^] ratio becomes oxidized. When the level of cytoplasmic lactate rises, it can inhibit ME1 activity [25], potentially unlinking these ratios and stimulating aging. The expression or injection of a bacterial lactate oxidase enzyme fused to catalase, which converts lactate to pyruvate, in aged humans, therefore, has the potential to alleviate reductive stress due to reduced [NAD^+^]/[NADH] to improve metabolism and potentially slow aging [22]. RNA-Seq studies of mice on a DR diet initiated at either 3 or 24 months of age have shown that the aged liver largely maintains the ability to transcriptionally adapt to DR, likely due to a healthy redox state, whereas aged adipose tissue fails to transcriptionally respond compared to the response of young control animals [26].

## 4. Small Increases in Cytoplasmic NADPH Are Hepatoprotective and Increase Health Span in Female Mice, While Larger Increases in WAT and Macrophage NADPH Stimulate Obesity and Inflammation

In most mammalian tissues, at least four enzymes are responsible for cytoplasmic NADPH generation, including ME1, isocitrate dehydrogenase 1 (IDH1), and the pentose phosphate pathway (PPP) enzymes glucose-6-phosphate dehydrogenase (G6PD) and 6-phosphogluconate dehydrogenase (PGD), while in the liver, the folate cycle enzymes MTHFD1 and ALDH1L1 are the primary cytoplasmic NADPH generators. The cytoplasmic [NADPH]/[NADP^+^] in WAT and the liver need to be tightly regulated for lipid synthesis. In rodent liver, the activities of ME1 and G6PD were shown to decrease after birth until 6 months of age, and then increase with aging for the rest of the lifespan [27,28]. The levels of hepatic cytoplasmic NAD^+^ kinase (NADK) that phosphorylates NAD^+^ to NADP^+^ were also shown to increase with aging [29]. These age-related increases in enzyme activities that contribute to cytoplasmic NADPH generation could offset the aging-related increase in mitochondrial superoxide generation and sulfhydryl oxidation to stabilize hepatic [NADPH]/[NADP^+^] with aging. Consistent with these activity changes being compensatory in response to increased ROS generation, DR decreased superoxide generation from mitochondrial electron transport chain (ETC) complex I [5,30,31] and largely prevented the aging-induced doubling of hepatic ME1 activity [32].

Although high levels of ME1 expression may improve the cytoplasmic redox state in the liver, ME1 expression in WAT is positively correlated with increased lipogenesis and adipocyte volume, consistent with a causative role in obesity [33]. There was increased G6PD expression in WAT when mice were fed a high-fat diet or in genetic models of obesity [34]. But one study found that WAT G6PD plus PGD activity decreased after only 8 weeks of a high-fat diet [35], which is consistent with another study that found an increased WAT NADH/NADPH after 4 weeks of high-fat diet treatment [36]. So, WAT cytoplasmic [NADPH]/[NADP^+^] may decline during the first couple of months of the high-fat diet in rodents during the prediabetes stage before it increases with continued high-fat diet consumption. G6PD and ME1 activities in WAT were not changed in obese humans compared to non-obese controls, although ATP-citrate lyase (ACLY) activity was increased [37]. However, diabetes led to a 50% decline in WAT ME1 activity. Lean, but not obese, diabetics showed a fourfold decline in WAT G6PD activity [38]. Constitutively increased NADPH levels that occurred as a result of global overexpression of G6PD in mice stimulated NADPH oxidase activity in macrophages and increased inflammation [39,40], while constitutively increased NADPH levels in white adipose tissue (WAT) stimulated excessive fatty acid synthesis, obesity [41], and type 2 diabetes [42,43]. In contrast, increasing NADPH by a more moderate, global twofold overexpression of G6PD using its endogenous promoter, or by nicotinamide riboside administration, protected female, but not male mice from hepatosteatosis [44], while slight G6PD overexpression also delayed aging-induced hearing loss in both male and female mice, although there was mild inflammation and cytokine increases in young mice [45]. Female G6PD transgenic mice also showed an extended median lifespan [46]. Increasing WAT ER [NADPH]/[NADP^+^] in mice via hexose-6-phosphate dehydrogenase (H6PD) overexpression increased WAT NADPH-dependent 11β-hydroxysteroid dehydrogenase type 1 activity and cortiocosterone production, leading to slight weight gain, glucose intolerance, and insulin resistance [47].

Branched chain amino acid (BCAA) supplementation was shown to decrease preadipocyte G6PD expression and NADPH levels to prevent obesity [48]. The mechanism through which NADPH stimulates obesity was shown to be through increasing the preadipocyte expression of the fat mass and obesity-associated (FTO) protein, which is an N^6^-methyladenosine (m^6^A) demethylase that demethylates the mRNA for cell cycle-stimulating proteins, preventing their degradation through an mRNA decay pathway, leading to increased numbers of preadipocytes and increased adipogenesis [49]. The overexpression of G6PD in 3T3-L1 preadipocytes stimulated the expression of adipocyte markers, increased triglyceride accumulation and the release of free fatty acids, increased the expression of proinflammatory cytokines such as TNF-α and resistin, decreased the expression of cytoprotective adiponectin, and stimulated insulin resistance [50]. Follow-up studies using isolated mouse adipocytes transduced with adenoviral G6PD confirmed the results in 3T3-L1 cells and further found that G6PD overexpression resulted in increased oxidative stress, leading to increased NF-κB activation. This increased the expression of NADPH oxidase subunits and increased proinflammatory cytokine release, ultimately stimulating the recruitment of monocytes to adipose tissue and increasing proinflammatory gene expression in macrophages that were incubated with the adipocytes [51].

Like G6PD in WAT, G6PD in macrophages was upregulated by proinflammatory stimuli, such as lipopolysaccharide and free fatty acids, and this led to oxidative stress and increased proinflammatory cytokine secretion [40]. G6PD mutant mice were protected from diet-induced obesity and insulin resistance. Importantly, the adoptive transfer of bone marrow from G6PD mutant mice to WT mice protected against diet-induced obesity and insulin resistance [52]. Therefore, many of the negative metabolic effects of increased G6PD activity in WAT could be reversed via G6PD-deficient macrophages. Under non-inflammatory conditions, a G6PD inhibitor did not alter NADPH levels in macrophages, suggesting that IDH1 [53] and ME1 [54] play important roles in the generation of macrophage NADPH in healthy cells, or at least they can be upregulated to completely compensate for the loss of G6PD. DR was shown to induce the expression of SIRT1 in macrophages, resulting in anti-inflammatory M2 polarization that was shown to be required for the DR-mediated browning of WAT [55].

Other data confirm the connection between increased cytoplasmic NADPH/NADP^+^ in lipogenic tissues and obesity. Mice overexpressing IDH1 have hyperlipidemia, fatty liver, and obesity [41]. The knockout of ME1, a major cytoplasmic NADP^+^-reducing enzyme in WAT [18] and a contributor to the reduction of cytoplasmic NADP^+^ to NADPH in the liver [19], led to decreased liver steatosis, fat mass, and obesity, and improvement in glucose tolerance [56]. Consistent with these data, single-cell RNA-Seq (scRNA-Seq) analysis showed that DR decreased the expression of NADK in several cell types in brown adipose tissue (BAT) and bone marrow, while DR also decreased the expression of nicotinamide phosphoribosyltransferase (NAMPT), the rate-limiting enzyme of NAD^+^ salvage synthesis, and PPP enzyme transoldolase (TALDO) in a cell population in WAT, which may lead to decreased NADP(H) levels and decreased NADPH oxidase activity, resulting in decreased inflammation. In contrast to its negative effects in macrophages and WAT during energy excess, G6PD appears to have positive effects on BAT metabolism. G6PD deficiency in BAT stimulated oxidative stress and inhibited cold-induced thermogenesis [57]. Flavonoids, such as quercetin, and catechin gallates, such as epigallocatechin gallate (EGCG) from green tea, have a high affinity for NADP(H) binding sites on proteins and strongly inhibit the activities of G6PD [58,59], NADPH oxidase [60,61], and cholesterol-synthesizing enzymes [62]. Therefore, some of the health benefits of tea consumption and plant-based diets may be through the action of these natural compounds decreasing NADP(H) metabolism in macrophages, WAT, and the liver.

## 5. Partial Loss of Function Polymorphisms in G6PD Are Common in the Human Population and Can Lead to Increased Longevity in Mice

Gene polymorphisms in the G6PD gene, present on the X chromosome, are the most common cause of enzyme deficiency in humans, present in 400 million people worldwide, 5% of the world population [63]. G6PD total loss-of-function mutations are embryonic lethal. However, individuals possessing at least 5% to 10% of normal enzymatic activity are born and possess varying levels of physiological dysfunction. The most common phenotype, when present, is anemia due to red blood cell (RBC) dysfunction. RBCs are the most susceptible cell type due to their loss of mitochondria, which serves as an important source of enzymes that reduce NADP^+^ to NADPH. G6PD loss of function was selected for in the human population due to the presence of the RBCs that are more resistant to malarial infection [63]. Surprisingly, G6PD-deficient mice were shown to have extended longevity [64], while some G6PD-deficient patients show protection from adverse cardiac events. It was hypothesized that the decreased NADPH from G6PD deficiency might decrease cholesterol synthesis and NADPH oxidase activity and H_2_O_2_ levels to decrease inflammation and mediate cardioprotection [65]. It would be informative to use tissue-specific G6PD-deficient mice to elucidate if decreased G6PD activity in macrophages, WAT, or other tissues drives the longevity benefit.

## 6. Fasting or DR Increased the Hepatic [NADPH]/[NADP^+^] and Reduced the Hepatic [NAD^+^]/[NADH]

Ground-breaking studies published in 1969 by Richard Veech and Hans Krebs showed that fasting for 48 h increased rat liver cytoplasmic (free) [NADPH]/[NADP^+^] from 70 before the fast to 177 after the fast. In this study the isocitrate/α-ketoglutarate ratio was measured as an estimate of the cytoplasmic [NADPH]/[NADP^+^] [30]. After correcting for tissue pH and tissue free Mg^2+^, the ratios increased to 99 before the fast and 226 after the fast [31]. Parallel uncorrected measurements of the malate/pyruvate as another measure of the cytoplasmic [NADPH]/[NADP^+^] showed an increase in the [NADPH]/[NADP^+^] from 85 before the fast to 538 after the fast [30]. For a discussion of the variability in these methods, see the following reference from John Williamson’s lab [66]. So, fasting increased the liver cytoplasmic [NADPH]/[NADP^+^] somewhere between 2.3 and 6.3-fold. Importantly, direct measurements of total NADPH and NADP^+^ showed a NADPH/NADP^+^ of 4.5 both before and after the fast, with the fast increasing both NADP^+^ and NADPH equally by 19%. So, direct measurements of NADP^+^ and NADPH levels do not yield an accurate assessment of the biologically important free levels. The reason stems from the fact that roughly 80% of intracellular pyridine nucleotides are bound to protein and not free in solution. Since NADP^+^ binds proteins with a higher affinity than NADPH due to its lower concentration [67], direct measurements do not yield information regarding the (free) [NADP^+^] and [NADPH] that control metabolism. A summary of the different results obtained when measuring the liver free cytoplasmic pyridine nucleotide redox ratios or liver total tissue pyridine nucleotide redox ratios with aging, DR, or fasting is shown in Table 1.

Follow-up studies from the Williamson lab confirmed the data obtained by Veech and Krebs and used the malate/pyruvate and reaction constants to calculate a hepatic cytoplasmic [NADPH] of 367 µM and a hepatic cytoplasmic [NADP^+^] of 4 µM ([NADPH]/[NADP^+^] = 92) during fed conditions that changed after 24 h of fasting to a cytoplasmic [NADPH] of 358 µM and a cytoplasmic [NADP^+^] of 2 µM ([NADPH]/[NADP^+^] = 179) [66]. These authors only found a 16% difference in the ratio when using the isocitrate/α-ketoglutarate instead of malate/pyruvate by using an improved experimental protocol for measuring the metabolite pair concentrations. Interestingly, these researchers calculated a highly oxidized hepatic mitochondrial [NADPH]/[NADP^+^] of 4.2 in fed rats and 3.4 in fasted rats. These mitochondrial [NADPH]/[NADP^+^] values are over 50-fold lower than the value of 219 obtained in cultured cells using a fluorescent biosensor. The biosensor measurements showed that the mitochondrial [NADPH]/[NADP^+^] was more than threefold more reduced than the cytoplasmic ratio of 65 in the cultured cells [71]. The reason for the disparate results using the different methods is unknown.

DR led to a 20% increase in the malate/pyruvate in the liver of 3-month-old mice and a 65% increase in this ratio in 30-month-old mice, following initiation of the diet at two months of age [72]. DR may increase cytoplasmic [NADPH]/[NADP^+^] in liver in part due to an increase in the level of cytoplasmic enzymes that reduce NADP^+^ to NADPH due to the hepatic activation of transcriptional regulators ATF4 [73] and Nrf2 (NFE2L2) [74], which induce the expression of several cytoplasmic enzymes that reduce NADP^+^ to NADPH, such as folate cycle enzymes. These transcriptional regulators can also function together, forming a heterodimer [75]. Another potential mechanism for increased hepatic cytoplasmic [NADPH]/[NADP^+^] during the DR diet is decreased lipid synthesis. In the liver of mice on a DR diet, one group showed decreased gene expression of the lipogenic transcriptional regulator liver X receptor α (LXRα) and its transcriptional target sterol regulatory element binding protein-1c (SREBP-1c) [76]. However, another group found that although DR upregulated the hepatic mRNA level of SREBP-1c in mouse liver, the abundance of its target proteins mediating lipogenesis was not altered [12]. Therefore, depending upon the fed or fasted condition or time of day when sacrificing the mice, DR may either decrease or not affect hepatic lipogenesis, while mRNA levels of lipogenic transcriptional regulators may even increase. The DR diet also decreased the gene expression of hepatic SREBP2 [77], a master transcriptional regulator of cholesterol synthesis, which could also contribute to the increased hepatic [NADPH]/[NADP^+^] and health benefits in DR mice.

In the case of a normal diet, rat liver cytoplasmic [NAD^+^] and [NADH] are roughly 500 µM and 1 µM, respectively, while mitochondrial values are roughly 4 mM and 500 µM, respectively [66]. Somewhat surprisingly, fasting caused a twofold reduction in hepatic cytoplasmic [NAD^+^]/[NADH], which is likely due to its link to the increased [NADPH]/[NADP^+^] [5,30,31]. A twofold reduction in cytoplasmic [NAD^+^]/[NADH] occurs in other peripheral tissues with aging, and this could potentially contribute to the increased susceptibility of these tissues to pathology and to the development of aging-related diseases [78,79]. During fasting, the liver may be more resilient to reduced [NAD^+^]/[NADH] due to the increased [NADPH]/[NADP^+^] and decreased oxidative damage.

## 7. Increasing the Generation of NADPH Extends Lifespan in *Drosophila*

High LDH and lactate levels, with relatively low cytoplasmic malic enzyme levels, are associated with aging in *Drosophila* [80] and mice [81], as a decreased pyruvate/lactate ratio leads to a reduced cytoplasmic [NAD^+^]/[NADH] ratio that disrupts metabolism in many tissues. Lactate also binds NADPH oxidase to stimulate ROS production and cellular senescence [72], while immune cell protein lactylation can lead to immunosuppression and increased tumorigenesis [82]. Overexpressing the cytoplasmic malic enzyme gene Men [83] in *Drosophila* extended lifespan [84,85], perhaps by linking the cytoplasmic [NAD^+^]/[NADH] and [NADPH]/[NADP^+^], buffering changes to these ratios. This link provides the benefit of channeling an aging-induced reduction in the cytoplasmic [NAD^+^]/[NADH], likely from decreased malate–aspartate shuttle (MAS) activity [86] and potentially from increased glyceraldehyde-3-phosphate dehydrogenase (GAPDH) expression [7] into increased cytoplasmic [NADPH]/[NADP^+^] to stimulate antioxidant defense and lipid synthesis. In mammals MAS activity likely declines with aging due to the decreased activity of its irreversible rate-limiting step, the mitochondrial electrogenic glutamate-aspartate exchanger (SLC25A12 and SLC25A13) [87,88], potentially as a result of decreased mitochondrial membrane potential. The increased GAPDH expression with aging in several rat tissues was also accompanied by the increased expression of the glycogen synthesis enzyme glycogenin 1 (GYG1), which together could potentially decrease NADPH-generating PPP flux, although the increased hexokinase 2 (HK2) expression that occurs in some aging tissues [7] could offset this effect to stimulate glycolytic and PPP flux. DR partially prevented the aging-induced increased expression of GAPDH, GYG1, and HK2, perhaps due to the DR-induced decrease in the expression of the glycolytic regulators HIF-1α and c-MYC [7]. A combination of cytoplasmic [NAD^+^]/[NADH] and [NADPH]/[NADP^+^] redox imbalances appear to contribute to most aging-related disorders [89]. More research is needed to determine the different dietary and physiological conditions that can modify the link between cytoplasmic [NAD^+^]/[NADH] and [NADPH]/[NADP^+^] and how the tissue-specific changes in these redox couples contribute to DR-mediated longevity.

## 8. DR Increases Hepatic ATF4 Activity That Increases Folate Cycle Flux to Increase NADPH Generation

Mitochondrial ETC activity declines with aging, resulting in decreased complex I-mediated oxidation of NADH and a reduced matrix space [NAD^+^]/[NADH]. The decrease in this ratio inhibits the mitochondrial folate cycle enzyme MTHFD2 and its paralog MTHFD2L [90], which primarily use NAD^+^ instead of NADP^+^ as a coenzyme due to the thousand-fold higher concentration of the former over the latter in the mitochondrial matrix. During fasting and DR, when glucose levels are low, the folate cycle can become an important source for the reduction of NADP^+^ to NADPH in cells that normally rely more heavily on PPP activity. MTHFD2 expression is regulated by the ATF4 transcriptional regulator. Increased folate cycle flux can increase cytoplasmic NADPH generation through the activity of the ALDH1L1 enzyme and in the liver, and perhaps other tissues, where folate cycle flux is reversed, through the activity of the methylene tetrahydrofolate dehydrogenase 1 (MTHFD1) enzyme [19]. ATF4 transcriptional activity can be downregulated via the inhibition of mTORC1 [91], which occurs under low nutrient conditions. But surprisingly, DR and other anti-aging interventions, such as protein or amino acid restriction [92], increase ATF4 activity in the liver [73]. Decreased cellular glucose levels or increased ER stress can also activate ATF4, increasing the expression of genes for cellular serine uptake and mitochondrial folate cycle genes, such as serine hydroxymethyltransferase 2 (SHMT2) and ALDH1L2. This leads to increased catabolism of serine and glycine, which stimulates folate cycle flux and the reduction of mitochondrial NADP^+^ to NADPH [93,94,95]. ATF4 expression is induced by Nrf2 [96]. A similar Nrf2-ATF4 transcriptional pathway appears to be activated after liver damage to generate the NADPH needed for deoxynucleotide and lipid synthesis for liver regeneration [97]. ATF4 also appears to be activated by DR in some cells in WAT, as scRNA-Seq experiments showed the ATF4 target genes ATF3 and DDIT3/CHOP are induced by DR in WAT [7].

A CRISPR/Cas9 gain-of-function screen was performed to determine the genes that, when overexpressed in a glucose-free, galactose-containing media, stimulate oxidative phosphorylation to prevent cell death due to a mutation of the ETC complex I subunit ND1 [90]. The top hit in the screen ME1 was found to restore cytoplasmic NADPH/NADP^+^ and glutathione (GSH)/glutathione disulfide (GSSG) and prevent cell death resulting from increased oxidative stress. So, aged cells with reduced mitochondrial [NAD^+^]/[NADH] may also have decreased cytoplasmic [NADPH]/[NADP^+^] due to decreased serine biosynthesis and catabolism decreasing folate cycle flux, at least in cells that heavily rely on oxidative phosphorylation for energy generation due to limited glucose metabolism (low glycolytic and PPP fluxes).

## 9. Some Tissues Possess a Reversible Mitochondrial Citrate-α-Ketoglutarate NADPH Shuttle, While a Citrate–Pyruvate Shuttle Is Present in Lipogenic Tissues to Provide Cytoplasmic Acetyl-CoA and NADPH for Lipid Synthesis

**Figure 1:**
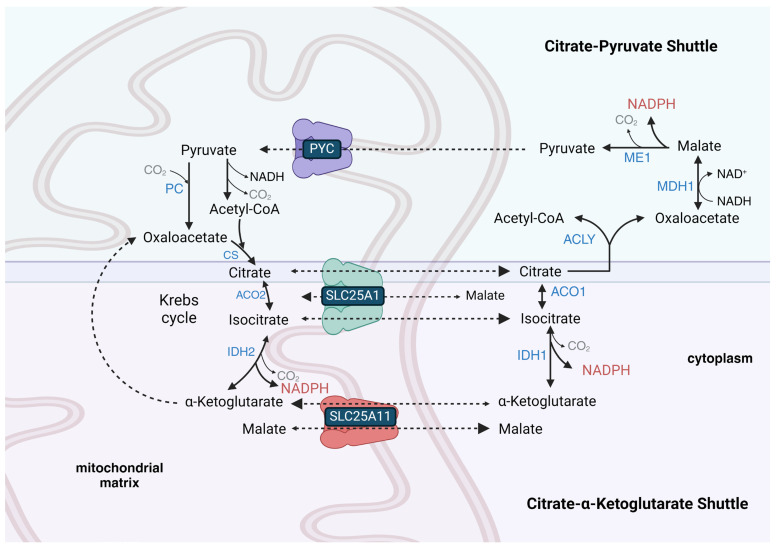
The citratrate-α-ketoglutarate shuttle and the citrate–pyruvate shuttle compete for cytoplasmic citrate. Both can generate cytoplasmic NADPH, but the citrate–pyruvate shuttle also synthesizes cytoplasmic acetyl-CoA and transfers cytoplasmic NADH-reducing equivalents into the mitochondrial matrix. The citrate–pyruvate shuttle relies upon pyruvate carboxylase (PC) to regenerate oxaloacetate to react with the pyruvate-derived acetyl-CoA. In the liver, both shuttles may operate simultaneously as the citrate–pyruvate shuttle can only provide roughly half of the NADPH required for fatty acid synthesis. In WAT, the remainder of the NADPH for fatty acid synthesis not provided by the citrate–pyruvate shuttle is likely synthesized by the PPP. Transport reactions and unnamed Krebs cycle reactions are shown as dashed arrows, while other chemical reactions are shown as solid arrows. Enzyme names are shown in blue font, metabolite names are shown in black font, coenzyme names are shown in maroon font, and transporter names and gaseous co-reactants and co-products are shown in gray font.

Some cell lines, such as HeLa (cervical cancer-derived) cells, have the ability to transport NADPH equivalents from the cytoplasm to the mitochondrial matrix [98], while others, such as HCT-116 (colorectal cancer-derived) cells, maintain independent cytoplasmic and mitochondrial NADPH metabolism [99]. This indicates that some cells do not possess an active mitochondrial citrate-α-ketoglutarate NADPH shuttle (Figure 1). This shuttle is also called the isocitrate-α-ketoglutarate shuttle [100]. The lack or restriction of its function in some cell types could be due to the limited activity of the SLC25A1 tricarboxylate (citrate and isocitrate) carrier protein that is mainly expressed in the liver, WAT, kidney, and pancreas, but is present at very low levels in skeletal muscle, heart, brain, and lung [101,102]. It could also be due to the limited transport of α-ketoglutarate into the matrix space that occurs in exchange for malate on the SLC25A11 carrier, which is mainly expressed in skeletal muscle, heart, liver, brain, and pancreas [103]. The mitochondrial isocitrate-α-ketoglutarate NADPH shuttle also relies on cytoplasmic IDH1 activity, which is expressed highly in the cytoplasm of the liver, heart, and skeletal muscle but at low levels in the adult brain. The shuttle further relies upon mitochondrial isocitrate dehydrogenase 2 (IDH2) activity, which is nearly ubiquitously expressed, with the highest expression in skeletal muscle, heart, liver, and kidney.

HCT-116 cells were shown to have unusual mitochondrial NADPH metabolism compared to most other cell lines, relying more highly on MTHFD2 and GLUD1 for the reduction of NADP^+^ to NADPH, with moderately low isocitrate dehydrogenase 2 (IDH2) expression and very low expression of NNT, malic enzyme 3 (ME3), and ALDH1L2 [104]. Across different cancer cell lines, cytoplasmic and mitochondrial [NADPH] were shown to positively correlate with NNT gene expression, while a negative correlation was found between mitochondrial [NADPH] and MTHFD1 expression [105], which is not surprising given that MTHFD1 oxidizes cytoplasmic NADPH in over half of all cell lines tested due to the specific direction of folate cycle flux established by the preferential serine catabolism by mitochondrial matrix SHMT2 compared to that of cytoplasmic serine hydroxymethyltransferase 1 (SHMT1) [19,106]. The enzymes involved in cytoplasmic and mitochondrial NADPH synthesis are shown in Table 2.

The mitochondrial isocitrate-α-ketoglutarate NADPH shuttle, unlike mitochondrial MAS [87], is reversible. During DR or fasting, this NADPH shuttle may function in the preservation of cytoplasmic [NADPH]/[NADP^+^] in cells that normally rely upon high glucose levels to drive PPP flux for the cytoplasmic reduction of NADP^+^ to NADPH. When glucose levels are low, TCA cycle-derived NADH, synthesized from mitochondrial-oxidized fatty acids and ketone bodies, can be used by mitochondrial NNT to synthesize NADPH. The mitochondrial citrate or isocitrate, derived largely from fatty acid, ketone body, and glucose catabolism during fasting, can be shuttled to the cytoplasm by SLC25A1 for IDH1 or ME1-mediated reduction of NADP^+^ to NADPH. The mitochondrial NADPH synthesized by the NNT can be oxidized to power the reductive carboxylation of α-ketoglutarate, where IDH2 reverses its normal direction, and the isocitrate produced is shuttled to the cytoplasm by SLC25A1 for the IDH1-mediated reduction of NADP^+^ to NADPH. In HeLa cells, the inhibition of SLC25A1 was shown to decrease cytoplasmic [NADPH] by 60% and mitochondrial [NADPH] by 47% [105].

The lifespan extension achieved via DR in C57BL/6J mice that lack the NNT [107] suggests that other mitochondrial enzymes that reduce NADP^+^ to NADPH or the mitochondrial isocitrate-α-ketoglutarate NADPH shuttle can cover for NNT loss, even during times of low food consumption. However, a co-expression analysis using hundreds of cell lines showed that NNT expression exhibited the highest level of co-expression with OXCT1, the gene encoding the rate-limiting enzyme of mitochondrial ketone body catabolism, suggesting the importance of NNT during fasting and DR [104]. Other enzymes that generate mitochondrial NADPH, as shown in Table 2, include ME3, GLUD1, and folate cycle enzymes ALDH1L2 and MTHFD2 [108]. Mitochondrial NADPH is needed for proline synthesis. So, cells severely deficient in mitochondrial NADPH synthesis, such as mitochondrial NAD kinase 2 (NADK2) mutants, are proline auxotrophs [109,110].

During times of reduced cytoplasmic [NAD^+^]/[NADH] when flux through glycolysis may be low due to the product inhibition of GAPDH, glutamine can be a carbon source used to synthesize citrate for a mitochondrial citrate–pyruvate shuttle-like metabolism, which transfers acetyl-CoA equivalents from the mitochondrial matrix to the cytoplasm for lipid synthesis, while also reducing one molecule of cytoplasmic NADP^+^ to NADPH and oxidizing one molecule of cytoplasmic NADH to NAD^+^. Before its carbons are fed into the shuttle as citrate, glutamine is transported from the cytoplasm into the mitochondrial matrix using the mitochondrial-targeted SLC1A5 glutamine transporter variant [111]. Mitochondrial glutamine is catabolized by glutaminase to glutamate, and then the glutamate is deaminated to α-ketoglutarate, which can then be reductively carboxylated to isocitrate, which is finally converted by aconitase 2 (ACO2) to citrate. As part of the shuttle, citrate is exported from the mitochondrial matrix space into the cytoplasm, where it binds ACLY along with coenzyme A to form acetyl-CoA and oxaloacetate (Figure 1). The oxaloacetate is used by malate dehydrogenase 1 (MDH1) to oxidize NADH to NAD^+^ with the linked synthesis of L-malate. The L-malate is decarboxylated to pyruvate with the concurrent reduction of NADP^+^ to NADPH. The cytoplasmic pyruvate synthesized by ME1 of the shuttle re-enters the mitochondrial matrix and is either decarboxylated to acetyl-CoA or carboxylated to oxaloacetate to maintain TCA cycle flux. The citrate–pyruvate shuttle can only generate roughly half of the NADPH required for fatty acid synthesis. So, the other half in WAT is likely derived from PPP flux and in the liver from the combination of serine catabolism with downstream folate cycle flux and IDH1 activity from flux through the isocitrate-α-ketoglutarate shuttle.

In mitochondrial ETC complex I mutant cells under low glucose conditions, the overexpression of ME1 enhanced the flux of cytoplasmic glutamine into the citrate–pyruvate shuttle for the cytoplasmic reduction of NADP^+^ to NADPH by ME1 [90]. Histone deacetylase 6 (HDAC6) inhibition was also shown to increase cytoplasmic citrate–pyruvate shuttle activity and the levels and activity of the shuttle enzyme MDH1 to increase cytoplasmic reduction of NADP^+^ to NADPH in liver-derived AML12 cells [112]. Consistent with this, DR largely blocked the 58% loss in hepatic MDH1 activity that occurred with aging [32], while DR also increased MDH1 expression in WAT [7]. During DR, the catabolism of glutamine for NADPH and lipid synthesis is likely increased in lipogenic tissues such as WAT [17] to compensate for decreased PPP activity, as there was shown to be increased NAD(P)H in WAT from aged DR mice compared to aged-matched controls [113]. Remarkably, removing glutamine from cell line culture media decreased the cytoplasmic and mitochondrial [NADPH] nearly as much as removing glucose (except for in HCT-116, where cytoplasmic [NADPH] was increased [104]), while glutamine supplementation increased NADPH/NADP^+^ and GSH/GSSG [114]. Glucose was also shown to be more important than glutamine for NADPH synthesis in neural progenitor cells (NPCs) [115]. Removing serine, another potential source of NADPH generation, from the culture media only led to small decreases in the cytoplasmic [NADPH] [104], except in MDA-MD-231 breast cancer cells, where it decreased by 31% [105].

## 10. PPP Enzymes G6PD and PGD Are the Major Reducers of Cytoplasmic NADP^+^ to NADPH in Many Cell Types and Tissues

NADP^+^ is mainly reduced to NADPH by the PPP enzymes G6PD and PGD in many cell types, such as T lymphocytes, and, to a lesser extent, red blood cells and neutrophils, but not in macrophages [116]. Likewise, PPP plays the major role in NADP^+^ reduction to NADPH in many tissues, such as the brain [117], BAT [19], thyroid, adrenal gland, ovaries, and testis [65]. Cytoplasmic G6PD activity is regulated by protein–protein interactions [118]. G6PD can be inhibited by its co-chaperone BAG3 when BAG3 is not phosphorylated [119]. BAG3 expression was shown to decrease with aging in rat liver, while DR was shown to decrease the expression of BAG3 in rat WAT to potentially stimulate G6PD activity [7]. G6PD can be activated by phosphorylated Hsp27 (HSPB1) [120]. While rat Hsp27 showed decreased expression with aging in five other tissues, including the liver, its expression in WAT increased with age. DR rescued these expression changes [7]. G6PD and PGD are greatly product-inhibited by NADPH under physiological conditions [121], but this product inhibition can be overcome through them binding the CRING protein, present only in lipogenic tissues [121] or by the HSCARG protein [121]. ME1 has also been shown to bind PGD, the third step of the PPP, and stimulate its activity to reduce NADP^+^ to NADPH to increase PPP flux [122], while malate, the substrate of ME1, had the opposite effect in terms of binding and inhibiting PGD activity [123]. For a more detailed discussion of PPP function, see the following review [124].

## 11. ME1 Together with PPP Enzymes Generate Most Cytoplasmic NADPH in WAT, While Folate Cycle Enzymes Predominate in Liver

Many cells and tissues do not rely on the PPP as the major cytoplasmic source for the reduction of NADP^+^ to NADPH. For example, in 3T3-L1 cells differentiated into white adipocytes, ME1 plays the largest role in the reduction of cytoplasmic NADP^+^ to NADPH under normoxic conditions, while the PPP played the major role under hypoxic conditions [18]. In WAT in vivo, ME1 and the PPP likely play roughly equal roles in the reduction of NADP^+^ to NADPH. ME1 functions directly after MDH1 in the mitochondrial citrate–pyruvate shuttle (Figure 1) [125]. The reliance of the citrate–pyruvate shuttle on mitochondrial oxidative metabolism explains the shift in NADPH generation to the PPP during hypoxia. ACAT1 (acetoacetyl-CoA thiolase), which catalyzes the final step of mitochondrial fatty acid beta-oxidation, also moonlights as a cytoplasmic acetyltransferase involved in the activation of ME1 by acetylating K337, which is antagonized by deacetylation by SIRT6 [126]. Surprisingly, ME1 knockout mice, even when knockout was initiated during adulthood, showed no observable phenotype when fed a normal diet [127], suggesting that IDH1 and PPP enzymes may be able to provide the cytoplasmic NADPH required for WAT lipogenesis. However, it is not yet known how the ME1 knockout mice respond to fasting–refeeding cycles or a high-fat diet.

**Figure 2:**
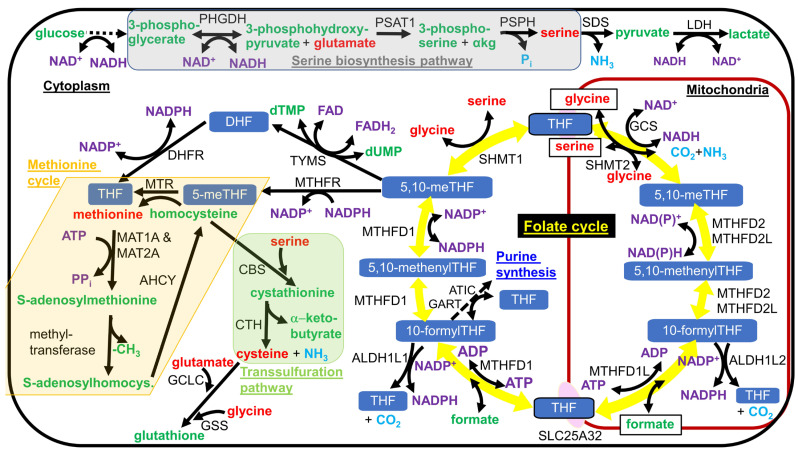
Serine biosynthesis pathway, the folate cycle, the methionine cycle, and the transsulfuration pathway. Serine is synthesized in a 3-enzyme pathway from the glycolytic intermediate 3-phosphoglycerate, as shown shaded in gray. In most tissues, with the known exception of liver, serine is imported into mitochondria and metabolized by SHMT2 to drive the folate cycle, shown as the circle of yellow arrows in the figure, in the clockwise direction. Formate, a one-carbon intermediate, and tetrahydrofolate are exported from mitochondria and react in the cytoplasm to from 10-formyltetrahydrofolate (10-formylTHF). Cytoplasmic one-carbon units can either be used for NADPH generation with the release of CO_2_ or in metabolism, where they are commonly used for methylation reactions or nucleotide synthesis. NADPH oxidation is used to regenerate cytoplasmic tetrahydrofolate (THF) from dihydrofolate. To be used for methylation reactions, the one-carbon unit is funneled into the methionine cycle, shown shaded as a light tan or cream color in the figure. Homocysteine can either be methylated to methionine and re-enter the methionine cycle or react with serine to enter the transsulfuration pathway, as shown shaded in light green in the figure, leading to cysteine and α-ketobutyrate synthesis. Different forms of folate are shown with a white font and blue background. Amino acid names are shown in red font. Other metabolite names are shown in green font, with the exception of coenzyme names that are shown in a purple font. Gaseous products are shown in light blue font. Enzyme names are shown in black font. Single chemical reactions are shown as solid arrows, while multiple reactions are shown as a dashed arrow. Metabolites that are transported into or out of the mitochondrial matrix are boxed.

As mentioned earlier, most hepatic cytoplasmic NADPH is synthesized by cytoplasmic folate cycle enzymes MTHFD1 and ALDH1L1 downstream of serine catabolism, fueling this metabolic flux [19] (counterclockwise flux through the folate cycle shown in Figure 2). ALDH1L1 knockout mice only showed a mild phenotype, consistent with the evolutionary loss of the ALDH1L1 ortholog in the common ancestor of reptiles and birds. The ALDH1L1 knockout mice showed mild glycine depletion in the liver due to slightly decreased cytoplasmic tetrahydrofolate levels [128]. In rhesus macaques, DR was shown to increase the hepatic acetylation of ALDH1L1 on K884 and increased the acetylation of the other folate cycle enzymes SHMT1 and SHMT2 on K416 and K464, respectively, which could potentially alter their enzyme activities and the rate of the reduction of NADP^+^ to NADPH [129]. In many mitotic cell lines in culture and in most postmitotic tissues, MTHFD1 consumes NADPH as part of the folate cycle (clockwise flux through the folate cycle in Figure 2).

IDH1 was shown to be the next most important enzyme for hepatic NADPH generation, having greater activity than ME1 or PPP enzymes G6PD or PGD, and was responsible for roughly 35–40% of cytoplasmic NADPH generation [19]. When high-dose acetaminophen was administered to hamsters to decrease hepatic GSH/GSSG, the level of MTHFD1 was unchanged, while there was a roughly 20% increase in IDH1 abundance [130]. So, IDH1 appears to play a larger role in the reduction of hepatic NADP^+^ to NADPH during mitochondrial stress that slows folate cycle activity. When examining global gene expression patterns in long-lived and short-lived strains of mice, IDH1 was identified as one of sixteen genes whose expression positively correlated with longevity [131].

The discovery that MAS activity is needed for the biosynthesis of serine [132,133], whose catabolism through the folate cycle is the major contributor to hepatic cytoplasmic NADPH generation [19], demonstrates another strong link between hepatic cytoplasmic [NAD^+^]/[NADH] and [NADPH]/[NADP^+^]. This link is due to the reduction of NAD^+^ by 3-phosphoglycerate dehydrogenase (PHGDH), the rate-limiting step of serine biosynthesis (Figure 2). When cytoplasmic [NAD^+^]/[NADH] is reduced, serine biosynthesis is slowed, and serine catabolism is shunted away from folate cycle flux toward pyruvate formation through the action of serine dehydratase (SDS), where LDH reduces pyruvate, while oxidizing NADH to restore cytoplasmic NAD^+^ levels. Through this mechanism, serine supplementation or PHGDH overexpression in the liver has been shown to activate SIRT1 and fatty acid beta-oxidation to prevent fatty liver [134]. Metabolic dysfunction raises S-adenosylhomocysteine levels that reduce flux through PHGDH, leading to redox imbalance [135], although the mechanism behind this effect has yet to be established. Increases in S-adenosylmethionine levels are known to inhibit methylene tetrahydrofolate reductase (MTHFR), leading to product inhibition of the two preceding MTHFD1-catalyzed steps and increased NADPH synthesis by ALDH1L1 [136]. Blocking the feedback inhibition of MTHFR by S-adenosylmethionine led to futile S-adenosylmethionine cycling, NADPH depletion, and folate cycle inhibition [137]. However, it is unclear if the folate cycle inhibition also feedback-inhibits the serine biosynthesis pathway since serine can be deaminated to pyruvate to bypass the folate cycle.

Glycine is reversibly metabolized to serine by cytoplasmic SHMT1 or mitochondrial SHMT2. Glycine supplementation extended the lifespan of mice [138,139], which may have partially been due to decreasing flux through the serine biosynthesis enzyme PHGDH and increasing flux from serine to lactate due to the product inhibition of SHMT1 by glycine, increasing cytoplasmic [NAD^+^]/[NADH]. Increased glycine or serine levels can also increase folate cycle flux, which may increase cytoplasmic and mitochondrial [NADPH]/[NADP^+^]. Therefore, serine or glycine supplementation can improve the cytoplasmic redox state, and this may be involved in the lifespan extension that occurs when either of these amino acids is supplemented to nematodes [140,141].

In pancreatic beta-cells, either ME1 or IDH1 is the primary generator of cytoplasmic NADPH, as an inhibitor of the mitochondrial pyruvate carrier (MPC) greatly slowed the reduction of NADP^+^ [142]. As shown in Figure 1, mitochondrial pyruvate is metabolized to acetyl-CoA and then to citrate, which is exported to the cytoplasm. If the citrate is metabolized by ACLY as part of the citrate–pyruvate shuttle, NADPH may be synthesized by ME1 [125]. If the cytoplasmic citrate is metabolized by aconitase 1 (ACO1), NADPH is likely synthesized by IDH1 as part of the citrate (or isocitrate)-α-ketoglutarate NADPH shuttle [100]. The citrate–pyruvate shuttle appears to be the more prevalent NADPH-generating pathway in pancreatic beta-cells following food consumption since IDH1 was shown to be a negative regulator of glucose-induced insulin secretion [100]. However, FGF21 addition, which mimics dietary protein restriction [143], was shown to increase pancreatic islet cytoplasmic NADPH levels by decreasing acetyl-CoA carboxylase-α (ACACA) levels, which decreased flux through the citrate–pyruvate shuttle (that includes ME1) linked to lipogenesis and increased flux through the citrate-α-ketoglutarate shuttle (that includes IDH1), which generates NADPH without providing acetyl-CoA for lipogenesis [144]. DR was shown to increase FGF21 signaling in rat WAT [145], which may also increase WAT cytoplasmic NADPH levels through increased flux through IDH1 to protect against aging-induced oxidative stress.

## 12. DR Increased the Activities of Cytoplasmic Enzymes That Reduce NADP^+^ to NADPH in Liver and WAT

Seven weeks of 50% DR in rats led to a twofold increase in the activities of liver cytoplasmic NADPH-generating enzymes ME1, G6PD, and PGD [146]. Another study using rats also found a twofold increase in liver ME1 activity upon refeeding for 48 h following long-term DR but not following short-term DR. In that study, WAT ME1 enzyme activity increased 15-fold upon refeeding following long-term DR, while ME1 activity did not change in any non-lipogenic tissue upon refeeding following DR [147]. Results from proteomics experiments of livers from DR mice did not find an increased abundance of PPP enzymes G6PD or PGD, but the abundance of ME1, IDH1, and the PPP enzyme transketolase (TKT) increased proportionally to the level of DR [148]. However, other investigators found no change in mouse hepatic ME1 levels during DR [12], suggesting that circadian rhythms may alter ME1 levels and activity. Euthanizing mice in either the fed or fasted state did not alter hepatic ME1 levels [12]. DR did not significantly alter the hepatic GSH/GSSG ratio in mice at 22 months of age [6], even though DR increased hepatic cytoplasmic [NADPH]/[NADP^+^] [5], showing that these ratios can be unlinked. GSH was shown to be secreted from the liver during methionine restriction to increase blood GSH levels and increase liver cytoplasmic H_2_O_2_ levels to activate Nrf2 [75,149]. It is unknown if this also occurs during DR to prevent the hepatic GSH/GSSG from increasing parallel with [NADPH]/[NADP^+^].

DR induced a fourfold increase in the gene expression of ME1 in WAT from mice as well as increased expression of the PPP genes TKT and TALDO [150]. Increases in gene expression of ME1 and TKT were also found at the protein level [9]. DR also prevented the aging-related increase in WAT aldose reductase (AKR1B1), a sorbitol pathway enzyme, which is a large consumer of NADPH when glucose levels are high [9]. Seven weeks of DR in rats increased the WAT activities of several cytoplasmic enzymes that reduce NADP^+^ to NADPH, including ME1, G6PD, and PGD by 3.4-fold, 5.4-fold, and 3.0-fold, respectively [146]. Consistent with this, there was an increase in NAD(P)H autofluorescence in the WAT of 30-month-old DR mice compared to control mice fed 95% of the amount of ad libitum-fed mice to prevent obesity [113]. Consistent with the suggestion that [NADPH]/[NADP^+^] in WAT or liver plays a role in longevity, overexpressing the *Drosophila* mitochondrial folate cycle enzyme Nmdmc, homologous to human genes MTHFD2 and MTHFD2L, specifically in the *Drosophila* fat body, the functional equivalent of mammalian WAT and liver, extended lifespan, while the expression of Nmdmc in the brain did not [151]. Increased folate cycle activity also plays an important role in nematode longevity, which appears to induce a common mechanism to that of methionine restriction [152]. Excitingly, both methionine restriction [153] and DR [17] increase WAT reduction of NADP^+^ to NADPH, lipogenesis, and fatty acid cycling in mice [154].

In mice, DR increased 2-[^14^C]2-deoxyglucose uptake in both perigonadal visceral WAT and inguinal subcutaneous WAT [155] due to increased Glut1 (SLC2A1) and Glut4 (SLC2A4) expression following feeding [145]. The increased 2-[^14^C]2-deoxyglucose uptake in WAT in DR mice supports the increased lipogenesis known to occur in WAT following each meal on the DR diet [17]. However, in contrast to DR, fasting was shown to decrease the expression and activities of WAT fatty acid synthesis enzymes ACLY, acetyl-CoA carboxylase-α (ACACA), and fatty acid synthase (FASN) [156,157,158]. It will be important to determine the molecular mechanisms through which DR and fasting have different effects on lipogenic gene expression in WAT and if there are conditions where the expression of WAT NADPH-generating enzymes diverges from the expression of the other lipogenic genes.

## 13. DR or Fasting Increases PPP Metabolites in the Plasma of Primates

Plasma metabolomics of DR in nonhuman primates, surprisingly, suggested increased PPP flux [159], although fasting is known to decrease PPP flux in the liver and skeletal muscle [160]. A 58 h fast in humans has also been shown to increase PPP metabolites in the blood [161]. The increased plasma PPP metabolites may be due to increased PPP flux in WAT from the increased glucose uptake mentioned above, but further experiments are needed to confirm this hypothesis, as DR also increased the expression of plasma membrane glucose transporter Glut1 (SLC2A1) in the skin [7]. Future studies should also measure PPP flux in WAT under feeding and fasting conditions in rodents, as 12 h of feeding after a 24 h fast in mice was shown to induce an increase in WAT fatty acid synthesis [19], so NADPH synthesis must have increased in parallel.

## 14. Full Lifespan Extension by DR Depends on Fasting for at Least 12 h and Feeding during the Active Portion of the Circadian Cycle

Pioneering studies showed that intermittent fasting without DR can extend the lifespan of mice by 11% [162,163]. Another study in which mice were fed via an automated feeding system was performed using five experimental groups that were all fed the same DR diet but were administered food at different times throughout the circadian cycle [77]. Mice that were administered their daily allotment of food at night, the active period for mice, over a 2 h or 12 h feeding period showed a 34% increased mean lifespan, while mice given their food over these durations in the daytime only showed a 20% extended mean lifespan. Mice given their DR diet spread out evenly over 24 h only showed a 10.5% extended lifespan. So, both a fasting period of roughly 12 h a day and feeding during the active phase of the circadian rhythm are essential for the full longevity benefits of DR.

The researchers performed RNA-Seq using the livers from the ad libitum and five different isocaloric DR diets described above [77]. They collected data every 4 h over the circadian cycle for both 6-month and 19-month mice to analyze changes with aging. Supplementary Table S1 shows circadian cycling and aging gene expression changes for the ad libitum and five DR diets for over 300 important genes and their transcriptional regulators, focusing on metabolism that may contribute to DR-induced longevity. Surprisingly, there was not a large general trend for loss of circadian oscillation with aging. Many genes even increased oscillation with aging. DR most frequently prevented circadian oscillations, but, in some cases, induced oscillations that were usually specific to one or two of the five different circadian DR feeding protocols. So, the data are complex, and it is very difficult to predict for any single gene how aging and DR affect the circadian regulation of hepatic gene expression. Notable observations were the loss of circadian cycling with aging for liver HDAC8, NCOR1, FOXO1, ACO1, and GSR, mitochondrial PDHB, CS, SLC25A51, and TXNRD2, and the lipogenic genes SREBP2, SLC25A1, ACLY, ACACA, and HMGCR [77]. It is important to perform a similar gene expression study using WAT and compare the results.

The liver aging and DR bulk RNA-Seq data also revealed many changes in the expression of genes regulating lipid droplet lipolysis that mostly are associated with decreased lipolysis with aging and increased lipolysis with DR [77]. Some lipid droplets associate with mitochondria and provide fatty acids for beta-oxidation [164]. DR decreased or prevented the aging-related increase in the expression of CIDEA, CIDEC (FSP27), G0S2, CLSTN3, PLIN2, and PLIN4 [77], while in BAT, DR increased the expression of SERPINF1 (PEDF), an activator of lipase ATGL (PNPLA2) [7]. In the WAT of DR mice, increases in hormone-sensitive lipase (LIPE) and lipoprotein lipase (LPL) were found at the protein level, as well as increased levels of fatty acid-binding protein 4 (FABP4). There was also an increased abundance of acyl-CoA synthetase long-chain family member 1 (ACSL1) and glycerol-3-phosphate dehydrogenase 1 (GPD1), which synthesizes the glycerol-3-phosphate backbone used for triacylglycerol synthesis [9]. The increased WAT lipolysis during the long fasting periods of the DR diet could increase free fatty acid and fatty acyl-CoA levels to inhibit acetyl-CoA carboxylase activity [165,166] and decrease the rate of fatty acid synthesis to increase cytoplasmic [NADPH]/[NADP^+^].

## 15. Feeding after Fasting on the DR Diet Increases WAT NADPH and Fatty Acid Synthesis Followed by Fatty Acid Oxidation Resulting in Fatty Acid Cycling

DR led to a roughly sevenfold increase in WAT GSH levels and GSH/GSSG [12]. The [NADPH]/[NADP^+^] has yet to be measured in WAT during DR. However, the large increases in the activities of enzymes that reduce NADP^+^ to NADPH [146], the large increase in the GSH/GSSG [12], and the increased NAD(P)H level [113] suggest that [NADPH]/[NADP^+^] increases as well, although this ratio may only increase transiently after feeding or fasting [167] or transiently throughout the circadian cycle [1,168].

In one important study, the DR diet greatly enhanced the daily oscillations in the rates of lipid synthesis and catabolism in WAT [17]. The DR mice in this study consumed all their daily allotted food, given at 6:00 PM, within 1 h each day, equivalent to the amount that the control mice consumed over an 8 h period. So, DR protocols in mice are typically intermittent fasting protocols [169]. Compared to control animals fed ad libitum, DR stimulated a threefold increase in the daily rate of fatty acid synthesis that occurred in WAT but not the liver. The rate of WAT fatty acid synthesis was greatly increased for 6 h after the initiation of feeding in a diurnal pattern, while daily whole-body fatty acid beta-oxidation rates were increased fourfold compared to controls, with major increases during the other 18 h of the day [17]. Subcutaneous WAT was particularly affected by the DR diet, showing a fourfold increase in fatty acid synthesis. The diurnal metabolic pattern was initiated one to two days after beginning the DR diet.

Strikingly, DR mice obtained 37% of their total energy needs from fatty acid oxidation, while control mice only obtained 7% [17]. The DR mice oxidized over 300 mg of fat a day but only consumed 92 mg of fat per day in their diet. So, roughly 70% of the fat they consumed each day was first synthesized in the WAT in the 6 h after their meal and then catabolized throughout the body for the next 18 h to yield a fatty acid cycling process. The DR mice lost weight only during the first week of DR treatment. After that, there was energy balance for the rest of the DR treatment [17]. Somewhat unexpectedly, the WAT from mice on the DR diet did not show increased monounsaturated fatty acid (MUFA) levels, although palmitic acid (16:0) levels increased and linoleic acid (18:2) levels decreased [17], which would decrease lipid peroxidation in adipocytes to inhibit ferroptosis [170]. Polyunsaturated fatty acids (PUFAs) from increased WAT lipolysis increase in the plasma during DR [171].

Others have shown that DR greatly decreased the membrane peroxidation index in many tissues. DR increased MUFA levels in the liver and kidney, while there were slight increases in the brain and heart but not in the skeletal muscle [172]. Phospholipids from isolated mitochondria showed similar changes as whole tissues, although mitochondrial membranes, in general, show a higher PUFA content [173]. These tissues also showed decreased n-3 PUFAs but not n-6 PUFAs. However, several of the tissues showed decreased levels of the highly peroxidizable n-6 arachidonic acid (20:4), with higher levels of the less peroxidizable n-6 linoleic acid (18:2) [172]. Therefore, it appears that the fatty acids synthesized in WAT, which are mostly comprised of saturated fatty acids and MUFAs, following a meal on the DR diet undergo lipolysis during the intermittent fast and are distributed to tissues throughout the body, where they both undergo beta-oxidation and are incorporated into lipid droplets and membrane phospholipids to replace PUFAs and decrease the membrane peroxidation index, potentially leading to decreased ferroptosis [174].

## 16. During DR, WAT SREBP-1c Induces Lipogenic Gene Expression That Is Required for DR-Mediated Longevity

The expression of SREBP-1c, a master regulator of lipogenesis, is stimulated by insulin [175] but inhibited by AMPK [176] and PUFAs [177]. In mice on the DR diet, three h after feeding, the gene expression of SREBP-1c was transiently increased in mouse liver and WAT, leading to a transiently increased gene expression of ACACA and FASN in the liver, but longer-lasting increases in ACACA and FASN gene expression in WAT. The increase in SREBP-1c in WAT was somewhat surprising given that carbohydrate response element-binding protein (CHREBP, also called MONDOB), encoded from the MLXIPL gene, had been described to be the major regulator of lipogenesis in WAT [178], but apparently not during feeding after fasting or DR, as confirmed in another report that monitored WAT ME1 levels, which increased in parallel with WAT SREBP-1c abundance when rats were fed ad libitum for 2 days following a DR diet [147]. Like in the liver, roughly 90% of WAT NADP^+^-dependent malic enzyme activity is composed of cytoplasmic ME1 activity [179]. Large increases in FASN protein levels were also measured in the WAT of mice on the DR diet [9]. Unlike the control animals, SREBP-1c knockout mice did not show an extended lifespan on the DR diet [180]. Decreased core body temperature, which also extends lifespan in mice [181], induces a similar lipogenic gene expression program in WAT that increases NADPH levels and supports WAT browning and thermogenesis [182].

Studies from the Partridge lab have shown that DR initiated at 24 months of age in mice failed to activate WAT SREBP-1c or induce the expression of its target genes ACLY, ACACA, FASN, and stearoyl-CoA desaturase 1 (SCD1). Therefore, the lifespan was not extended. The Gene Ontology term “NADP metabolic process” showed the fifth highest score for genes that were induced by DR in WAT of young but not aged mice. An altered gene expression of preadipocytes due to increased inflammation in the aged animals was shown to be the likely cause for the lack of transcriptional plasticity in the aged WAT. Peroxisomal proliferator receptor coactivator-1α (PGC-1α)-dependent gene expression involved in WAT browning was also not strongly induced in the mice when the DR diet was initiated at 24 months of age compared to its strong induction by DR in young animals [26], supporting data that SREBP-1c functions upstream of PGC-1α in WAT [12]. Similar findings with fasting and refeeding, instead of the use of a DR diet, were found between 2-month and 22-month rats, where refeeding a high-carbohydrate diet, after 3 days of fasting, induced G6PD, ME1, ACLY, and FASN activities nearly sixfold in the young rats but only twofold in the aged rats [183]. Refeeding a high-fat diet to the young animals only led to a slight increase in the activities of G6PD and ME1 [184], as expected due to the known regulation of WAT SREBP-1c by insulin [175]. The failure of DR to induce lifespan-extending lipogenic gene expression in aged WAT may be in large part due to the aging-related decrease in gene expression of many transcriptional regulators required for the clonal expansion of preadipocytes or their differentiation into adipocytes such as KLF4, C/EBPβ, C/EBPδ, STAT5A, and the AP-1 subunits JUN, JUNB, and FOS, while DR initiated from youth blunted the aging-induced decreased expression in WAT KLF4, C/EBPβ, C/EBPδ, and FOS [7,185]. The expression of the YBX1 RNA-binding protein required for autophagy and adipogenesis [186] also declined in abundance with aging in WAT, and this decline was blunted by the initiation of DR in youth [7].

In the liver, the major transcriptional inducers of lipogenesis are SREBP-1, CHREBP, liver X receptor α (LXRα/NR1H3), and liver X receptor β (LXRβ/NR1H2) [178]. LXRα induces the expression of SREBP-1c and FASN [178]. Fasting leads to LXRα phosphorylation in the liver, inhibiting its DNA binding and activity [187]. LXRα plays an opposite role in WAT, stimulating lipolysis and fatty acid beta-oxidation [188]. In WAT, DR was shown to increase the expression of LXRβ [7]. In mouse liver, the gene expression of both SREBP-1c and CHREBP declined with aging, while DR only prevented the decline in CHREBP expression [77]. However, DR prevented the aging-induced decline in the expression of SREBP cleavage-activating protein (SCAP) [77], a cholesterol-activated chaperone required for SREBP ER to Golgi transport, stimulating SREBP cleavage and release from the Golgi for its nuclear translocation [189]. So, DR has the potential to partially blunt the loss of functional hepatic SREBP-1c protein that may occur with aging. Hepatic SREBP-1c induces the gene expression of cytoplasmic enzymes that reduce NADP^+^ to NADPH, including G6PD, PGD, and ME1, as well as FASN and GSH-synthesizing enzyme GCLC [190]. In the liver of DR mice, SREBP-1c gene expression is activated following meal consumption. SREBP-1c gene expression peaked 3 h after feeding and then declined during the rest of the day while fasting, decreasing to roughly 30% of that of ad libitum-fed controls just before the animals were fed the next day. Other studies found that fasting decreased hepatic SREBP-1c transcriptional activity [191,192] or DR decreased hepatic lipogenic gene expression [193]. In contrast to the gene expression data, liver protein levels of the SREBP-1c targets ME1, ACLY, ACACA, and FASN did not change in response to DR [12], and hepatic fatty acid synthesis rates, measured by heavy isotope (^2^H_2_O) labeling and mass spectrometry, did not differ between ad libitum and DR mice [17]. It is unclear why the DR-induced hepatic changes in lipogenic gene expression are not seen at the protein level.

In mice on the DR diet, WAT SREBP-1c gene expression increased transiently after feeding, after which it declined back to the level found in the ad libitum-fed controls [17]. SREBP-1c transcriptional activity in the liver is normally activated by feeding [12], most highly after a period of fasting or DR [191], but in WAT, it only appears to be induced by feeding after fasting, especially after long-term DR. Importantly, unlike the control mice, SREBP-1c knockout mice on a DR diet showed greatly blunted induction of WAT cytoplasmic enzymes that reduce NADP^+^ to NADPH as well as other enzymes involved in lipogenesis such as FASN. JAZF1 is a negative transcriptional regulator of SREBP-1c [194]. In one study, DR was shown to decrease the hepatic gene expression of JAZF1 [77], while in another study, aging decreased hepatic JAZF1 expression [194], which could contribute to the aging-induced increase in lipogenesis that occurs in early and mid-adulthood [195]. Lastly, SREBP-1c has been shown to have a transcription-independent role in WAT by binding (poly(ADP-ribose) polymerase 1 (PARP1) to stimulate DNA repair, delaying cellular senescence and inflammation. A high-fat diet was shown to decrease WAT SREBP-1c levels and stimulate WAT cellular senescence [196]. As DR also delays cellular senescence [197,198], it will be important to determine if the transcription-independent role of WAT SREBP-1c plays a role in the DR-mediated delay in cellular senescence and increase in longevity.

It will also be important to determine further molecular mechanisms involved in the regulation of WAT SREBP-1c transcriptional activity, including if SIRT1-mediated deacetylation and inactivation of SREBP-1c occurs during fasting in WAT as it does in the liver [199,200], if this is reversed by refeeding, and if it is altered by a DR diet. The increased NAD^+^ levels in WAT during fasting activate SIRT1 and SIRT2 to inhibit adipogenesis through the deacetylation and activation of FOXO1, which inhibits PPAR-γ activity, leading to decreased SREBP-1c and FASN levels [201,202]. Future studies could determine if this also occurs during the fasting period of the DR diet. Some SIRT1 expression is required to decrease oxidative stress and allow adipogenesis in WAT to occur [203]. So, studies are needed to determine the effects of a DR diet on the circadian changes in [NADPH]/NADP^+^] and in the levels and activities of enzymes that reduce NADP^+^ to NADPH in WAT. DR may increase WAT [NADPH]/[NADP^+^] to a small extent, similar to the twofold increase in G6PD levels that led to extended health span, while high-level constitutive overexpression of an enzyme that reduces NADP^+^ to NADPH in WAT has been shown to lead to larger constitutive increases in [NADPH]/[NADP^+^], resulting in excessive fat accumulation, inflammation, and metabolic disease [41], likely resulting in decreased longevity.

## 17. WAT SREBP-1c May Signal in Part through FGF21 for DR-Mediated Mitochondrial Biogenesis and Longevity in Male Mice

DR decreased WAT leptin autocrine signaling to increase SREBP-1c expression, which induced the expression of FGF21 and PGC-1α, leading to increased WAT mitochondrial biogenesis in male mice [180]. DR mice that were fed prior to euthanasia also showed increased WAT expression of CHREBP and PPAR-γ, which function together with SREBP-1c to induce the expression of WAT FGF21 [145]. DR-induced changes in energy balance and body composition were largely independent of FGF21 in mice, but WAT browning was dependent on FGF21 in male but not female mice, while a lack of FGF21 blunted DR-mediated changes in glucose tolerance and insulin sensitivity in female mice [204]. Others have also found large sex differences in how DR affects WAT in mice [205]. FGF21 was required for the lifespan extension induced by a protein-restricted diet in male mice [206], and the transgenic overexpression of FGF21 extended the lifespan of ad libitum-fed male mice [207]. FGF21 secreted from WAT is thought to function as an autocrine signal and does not appear to greatly influence systemic FGF21 levels that are primarily controlled through hepatic FGF21 secretion. DR was shown to increase the expression of FGF21 in WAT but did not increase FGF21 levels in the liver or plasma. Hepatic FGF21 is induced by protein restriction but not by carbohydrate restriction [208], although prolonged fasting can induce the expression of hepatic FGF21 in a PPAR-α dependent manner [209]. One study showed that DR prevented the aging-related increase in hepatic FGF21 gene expression [77], while another study showed that plasma FGF21 levels increased with aging and in mice that were fasted prior to euthanasia, DR prevented this increase, but not in mice that were fed prior to euthanasia [145].

There are three members of the NR4A group of nuclear receptors that transcriptionally regulate many genes involved in metabolism: NR4A1 (Nur77), NR4A2 (Nurr1), and NR4A3 (Nor1). The expression of rat NR4A1 increased with DR in WAT, while the expression of WAT NR4A2 and NR4A3 decreased [7], while others have shown NR4A1 expression to be increased in the liver and skeletal muscle in response to DR [210]. Increased NR4A1 expression decreases inflammatory markers [211], and NR4A1 protein levels decrease with aging [212], possibly contributing to the increased inflammation that occurs with aging. NR4A1 stimulates mitochondrial biogenesis and fatty acid beta-oxidation [213]. There is increased hepatic NR4A1 expression with prolonged fasting, and this was required for increased hepatic FGF21 expression [214]. The stable overexpression of NR4A1 in preadipocytes in cell culture stimulated lipolysis [215]. Consistent with this, female NR4A1 knockout mice fed a high-fat diet showed increased weight and fat mass [216]. In 3T3-L1 preadipocytes, NR4A1 expression was induced by insulin or PPAR-γ agonists [217]. NR4A1 was required for the fasting-induced repression of WAT PPAR-γ expression [218]. NR4A1 expression in BAT induced uncoupling protein 1 (UCP1) expression and thermogenesis [219]. The increased expression of NR4A1 in the liver inhibited SREBP-1c and lipogenesis [220]. The data suggest that interventions that increase WAT, BAT, and liver NR4A1 levels are potential therapies for aging-related metabolic disorders [221].

## 18. Different WAT Depots Possess Different Respiratory Rates and during DR Show Slightly Different Responses in Lipogenic Gene Expression

Studies by the Higami lab have shown that the levels of ME1 and ACLY, both SREBP-1c target genes functioning in lipogenesis, are coordinately regulated in two groups of DR rats that differed only in that one group was fed before sacrifice (DR-fed rats), and the other group was fasted before sacrifice (DR-fasted rats). In DR-fed rats (DR rats that were fed 16 h before they were euthanized) and DR-fasted rats (DR rats that were fasted for 24 h before they were euthanized), there were slightly different effects on the abundance of ME1 and ACLY in three different WAT depots, including retroperitoneal WAT (rWAT), epididymal WAT (eWAT), and subcutaneous WAT (sWAT). ME1 levels were higher in all DR samples, but with DR-fed rats having 2.4-fold, 3.5-fold, and 1.4-fold higher ME1 levels than DR-fasted rats in rWAT, eWAT, and sWAT, respectively. ACLY levels increased in parallel, with DR-fed rats having 3-fold, 5-fold, and 2.5-fold higher levels than DR-fasted rats in rWAT, eWAT and sWAT, respectively. Since fatty acid synthesis enzymes increase in parallel with ME1, there may or may not be a net increase in the [NADPH]/[NADP^+^] in WAT during DR, under either the DR-fed or DR-fasted conditions, as NADPH could potentially be oxidized by FASN at the same rate it is synthesized. Malate/pyruvate measurements to estimate the cytoplasmic [NADPH]/[NADP^+^] in WAT are therefore needed in DR animals at several different time points throughout the circadian cycle, or at least in DR-fed and DR-fasted animals to determine the daily dynamics.

Different adipose tissue depots were found to have different mitochondrial respiratory rates with BAT having, by far, the highest, followed by gonadal WAT in females, but gonadal WAT and inguinal WAT showed roughly equal rates in males, and with mesenteric WAT showing the lowest respiratory rate [222]. It will be interesting to determine the possible differential effects of DR on the browning of these different WAT depots. Data from mice partially addressing this issue showed that DR induced the browning of both visceral and subcutaneous WAT [55].

## 19. The *C. elegans* Homolog of SREBP-1 Functions to Promote Longevity by Increasing the Level of MUFAs

SBP-1, the *C. elegans* homolog of SREBP-1, also functions in extending lifespan [223,224]. Much of the pro-longevity benefit is mediated via the induction of the transcription of Δ9 fatty acid desaturases *fat-5*, *fat-6*, and *fat-7*, homologous to human SCD and SCD5, which synthesize MUFAs. The transcriptional regulator PHA-4, homologous to mammalian FOXA1, FOXA2, and FOXA3, is required for DR-mediated longevity and also induces the expression of these fatty acid desaturases [225]. Consistent with this, FOXA1 and FOXA2 functions in WAT have been implicated in mediating some of the protective metabolic effects of DR [226], although the *Drosophila* homolog of FOXA was shown to extend lifespan through its function in the intestine [227]. Specifically, FOXA1 has been shown to increase the expression of LXRα in hepatocytes, which regulates systemic insulin levels [228], while FOXA1 also stimulates pancreatic glucagon expression [229].

The inhibition of the *C. elegans* COMPASS histone H3K4 trimethylation complex led to SBP-1 nuclear translocation and MUFA synthesis, extending lifespan [223]. When glucose was added to *C. elegans* culture, SBP-1 and the mediator complex subunit MDT-15 [230] were activated downstream of the transcriptional regulator MXL-3, a homolog of human MAX protein, stimulating lipogenic gene expression [231]. In the case of *C. elegans* in the intestine, like in the mammalian liver, fasting activated SIR-2.1, homologous to mammalian SIRT1, which deacetylated and inactivated SBP-1 [200]. SBP-1 was shown to induce the expression of many one-carbon metabolism genes, including genes of the methionine cycle and the transsulfuration pathway (Figure 2). Related to this, SBP-1 was shown to be activated when methionine cycle gene S-adenosylmethionine synthetase-1 (*sams-1*), homologous to human MAT1A and MAT2A (shown in Figure 2), was knocked down, which led to increased longevity and the partial restoration of phosphatidylcholine synthesis [232,233].

SBP-1, DAF-16 (homologous to mammalian FOXO proteins), and DAF-7 (homologous to mammalian TGF-β) were shown to be particularly important for the lifespan extension that occurs as a result of the synergy among different pro-longevity drugs that work together to stimulate MUFA synthesis [234]. The MUFA oleic acid (C18:1) appears to extend lifespan by increasing the number of lipid droplets that activate the endoplasmic reticulum (ER)-associated degradation (ERAD) machinery, stimulating the processing of ER-localized Nrf1 homolog SKN-1A, inducing its nuclear translocation [235]. Increased peroxisomal number and decreased membrane lipid peroxidation to prevent lifespan-shortening ferroptosis [236,237] were also involved [238]. The compound dioscin, a natural saponin glycoside, activated XBP-1, homologous to human XBP1, activating the ER stress response, which activated SBP-1 in the intestine, leading to the expression of FAT-5 and FAT-7, culminating in lifespan extension [239]. Dietary MUFAs could rescue the reduced longevity of *sbp-1* or *xbp-1* mutants. Dioscin apparently activated the ER stress response through XBP-1 in neurons that signaled for increased intestinal *sbp-1* expression, increasing MUFA levels and SKN-1A transcriptional activity to extend lifespan.

## 20. CHREBP Induces the Expression of NADPH-Generating and Other Lipogenic Genes and Its *C. elegans* Ortholog MML-1 Is Required for DR-Mediated Longevity

In addition to SREBP-1c, CHREBP is another transcriptional inducer of lipogenic gene expression. CHREBP is highly expressed in the liver, WAT, and BAT and may play a role in DR-mediated longevity in WAT and the liver [240], although this has yet to be firmly established. Hepatic ME1 levels were decreased by 59% in CHREBP knockout mice, the greatest decrease in any lipogenic gene measured, leading to a 65% decrease in fatty acid synthesis [241]. Histone deacetylase 3 (HDAC3) knockdown was shown to increase the gene expression of CHREBP in adipocytes, associated with increased PGC-1β expression, mitochondrial gene expression, and browning of WAT [242]. WAT-specific knockout of CHREBP completely blocked sucrose-induced de novo lipogenesis in WAT and stimulated insulin resistance in WAT, the liver, and muscle [243]. The data suggest that SREBP-1c and CHREBP function together in WAT during the DR diet to induce the genes needed for lipogenesis following meal consumption.

There appears to be an evolutionarily conserved ability of increased NADPH and fatty acid synthesis to extend longevity, as the CHREBP (and MYC) homolog in *C. elegans* MML-1 and its binding partner MXL-2 (homologous to human MLX) are required for several different nematode paradigms of lifespan extension, including DR, decreased insulin signaling, germline removal, or decreased TOR signaling. MML-1 but not MXL-2, was partially required for the lifespan extension induced via the activation of the mitochondrial unfolded protein response (UPR^mt^) [244,245]. MML-1 functions together with HLH-30, homologous to mammalian TFEB, a master transcriptional regulator of lysosomal and autophagosomal activity to extend lifespan [245]. MML-1 was shown to function in neurons and hypodermis to extend the lifespan of a germline-defective *glp-1* mutant strain [246], while MXL-2 was only required in neurons. Fasting was shown to induce the translocation of intestinal MML-1, together with HLH-30, from the nucleus to the cytoplasm [247], consistent with it functioning in intestinal nuclei, to induce the expression of genes that catalyze the conversion of glucose to fatty acids after feeding. In stark contrast to this result, DR or reduced insulin signaling was shown to increase the translocation of intestinal MML-1 from the cytoplasm to the nucleus, which was dependent upon the transcriptional regulator PHA-4, but not DAF-16 [244]. MML-1 functions together with the ER stress response transcriptional regulator XBP-1, which induces the expression of fatty acid desaturase for the synthesis of the MUFA oleic acid [248], to extend longevity [235], as outlined above.

The *C. elegans* H_2_O_2_-generating NADPH-dependent BLI-3 dual oxidase, homologous to human DUOX1 and DUOX2, is present in both the hypodermal plasma membrane for tyrosine crosslinking in collagen for cuticle synthesis and in the intestine for the activation of SKN-1/Nrf [249]. MLT-7 peroxidase forms a complex with BLI-3. The knockdown of *mml-1* in neurons led to decreased global *mlt-7* expression. The global knockdown of *bli-3* or *mlt-7* blunted lifespan extension in the long-lived *glp-1*-deficient germline-defective animals and knocking down *mlt-7* specifically in the intestine phenocopied this effect [246]. This suggests that in response to germline removal, neuronal MML-1 signals to the intestine to increase the expression of MLT-7, which prevents excessive intestinal H_2_O_2_ that allows for balanced SKN-1/Nrf activation and lifespan extension [250]. Consistent with its role in the synthesis of fatty acids from glucose, neuronal *mml-1* knockdown led to the decreased expression of plasma membrane glucose transporter *slcf-2*, mitochondrial TCA cycle gene citrate synthase (*cts-1*), and citrate–pyruvate shuttle component ATP-citrate lyase (*acly-1*) [246].

## 21. Transcriptional Regulators FOXO1 and FOXO3 Induce the Expression of Genes That Reduce NADP^+^ to NADPH, While FOXO3 Is Required for DR-Induced Longevity

Reduced insulin signaling extends the lifespan in invertebrates through an AKT-mediated signaling pathway that results in the nuclear translocation of FOXO orthologs that mediate a pro-longevity transcriptional response [155]. Mammals have four FOXO paralogs: FOXO1, FOXO3, FOXO4, and FOXO6. Polymorphisms in the FOXO3 gene are associated with extended human longevity [251]. Constitutively activating FOXO3 in the liver was shown to upregulate G6PD gene expression, leading to increased NADPH/NADP^+^ [115]. Glutathione peroxidase expression was also increased. FOXO3 directly stimulated the expression of SREBP-1c in the liver [252], while in adipocytes, FOXO3 stimulated autophagy that resulted in acetyl-CoA for lipogenesis and fat accumulation [253,254]. FOXO3-deficient neural stem cells showed deficient glutaminolysis, an important source of NADPH synthesis, due to reduced glutaminase and GLUD1 activity. These cells also showed decreased GSH synthesis as well as decreased PPP flux due to decreased PGD expression, leading to increased ROS levels [255]. In human retinal epithelial cells, the knockdown of FOXO1 or FOXO3 decreased IDH1 gene expression and decreased NADPH/NADP^+^, leading to a small compensatory increase in IDH2 and ME1 gene expression without affecting G6PD expression [255]. In contrast to FOXO3, FOXO1 represses hepatic SREBP-1c expression and fatty acid biosynthesis [256,257]. Like FOXO1 knockout, triple liver-specific knockout of FOXO1, FOXO3, and FOXO4 redirected metabolic flux from gluconeogenesis to lipogenesis [258]. FOXO3 was shown to be required for the lifespan extension induced by DR in mice [259], while FOXO1 was shown to be required for the tumor suppression induced by DR but not for the longevity effect [260]. A link between WAT FOXO activity and longevity comes from the extended lifespan of the fat-specific insulin receptor knockout (FIRKO) mice [261].

## 22. Decreased Hepatic ETC Complex I Activity Stimulates the Reduction of WAT NADP^+^ to NADPH and Fatty Acid Cycling in Several Extended-Longevity Paradigms

The von Zglinicki group made a breakthrough in determining the mechanism of DR-mediated longevity when they discovered that there was decreased ETC complex I-mediated superoxide production due to the decreased abundance of several ETC complex I membrane arms subunits such as NDUFA5, NDUFS1, NDUFS2, NDUFS3, and NDUFV2 in the livers of DR mice [8]. The brains taken from DR mice also showed a decreased abundance of several complex I membrane arm subunits. Earlier studies from the Barja group found decreased complex I content in DR mice [262]. Complex I abundance or membrane arm subunit levels were also decreased in hearts taken from ad libitum-fed pigeons, a long-lived species compared to rats that have a similar body mass but are short-lived [263], and for other longevity therapies, such as rapamycin treatment [8], protein restriction [264], and methionine restriction [265]. For a review, see [266]. Longevity-promoting methionine restriction was shown to decrease fatty acid synthesis in the liver and increase fatty acid synthesis and beta-oxidation in WAT to activate interorgan fatty acid cycling [153]. Fatty acid beta-oxidation increased by 40% in the liver and fivefold in WAT, which matched the fivefold increase in mitochondrial mass in WAT, as estimated by citrate synthase activity.

The administration of ETC complex I inhibitors, such as the diabetes drug metformin or the Parkinson’s disease toxin 1-methyl-4-phenyl-1,2,3,6-tetrahydropyridine (MPTP), led to fatty acid cycling [154]. It was hypothesized that mitochondrial complex I inhibitors are prevalent in plants and microbes in the vertebrate diet that affect the intestinal and hepatic mitochondrial ETC, but spare complex I in WAT. So, vertebrate organisms may have evolved fatty acid cycling as a mechanism to minimize flux through intestinal and hepatic ETC complex I and maximize flux through ETC complex II. This altered metabolism minimizes ETC-mediated superoxide generation in aged organisms [154] since complex I is the major site of increased ROS production with aging [266]. The inhibition of hepatic mitochondrial transcription appears to decrease complex I activity to also induce this metabolically protective effect [267]. Synthesizing fatty acids in WAT and oxidizing them in the liver establishes a metabolically futile but redox-active cycle, where NADPH synthesized in WAT is effectively shuttled into FADH_2_ in the liver to bypass ETC complex I dysfunction, with no net effect on NADH levels, but greatly decreased ATP levels. The researchers named this cycle the “NADPH-FADH_2_ axis”. DR likely activates the NADPH-FADH_2_ axis due to the decreased hepatic ETC complex I membrane arm subunit levels and complex activity that may communicate with WAT through mitokines such as GDF15. GDF15 has been shown to be a marker of mitochondrial dysfunction in WAT. GDF15 gene expression increased in aged human subcutaneous WAT, which correlated with decreased lipogenesis in the elderly [268]. The expression of GDF15 decreased in WAT in rats on a DR diet, suggesting improved mitochondrial function [7].

Due to the loss of ATP, the NADPH-FADH_2_ axis may be most active during the first week or so of DR, when there is a negative energy balance and weight loss. Reverse electron transport from complex II through ubiquinone to complex I can also be a source of superoxide generation that increases during fatty acid cycling. However, increased superoxide generation from reverse electron transport in *Drosophila* was shown to be associated with increased longevity [269], and in mice, DR was shown to decrease reverse electron transport [270], most likely through the decreased abundance of the complex I membrane arm subunits [8]. It was hypothesized that the fatty acids synthesized by WAT could be oxidized in the liver and that the liver could release glucose, lactate, and ketone bodies, which are taken back up by WAT for fatty acid synthesis [154].

## 23. Does DR-Induced Expression of SREBP-1c Decrease WAT [NADPH]/[NADP^+^] Contributing to the Metabolic Benefits of DR?

It is possible that SREBP-1c activation in WAT during DR decreases [NADPH]/NADP^+^], leading to beneficial metabolic effects. This could occur if SREBP-1c activation leads to a proportionally higher increase in the activity of the enzymes that convert citrate and acetyl-CoA to fatty acids than the activity of NADPH-generating enzymes required to support that increased metabolic flux. Consistent with this hypothesis, adipose-specific knockout of the PPP enzyme TKT protected mice against the harmful effects of a high-fat diet. It was shown that these mice had decreased WAT NADPH levels and decreased glycolytic flux and pyruvate levels [271]. The decreased glycolytic flux may be due to increased H_2_O_2_ levels that oxidatively inactivate glycolytic enzymes [272]. The decreased glycolytic flux activates WAT lipolysis and fatty acid beta-oxidation for energy generation [271]. Unexpectedly, TKT knockout in BAT stimulated thermogenesis, in part through increased glucose uptake by increasing Glut4 levels, decreasing the levels of TXNIP, an inhibitor of Glut4 expression [273]. WAT-specific knockout of mitochondrial SOD2 also led to increased mitochondrial biogenesis, UCP1 expression, and protection against a high-fat diet [274,275], consistent with the involvement of increased WAT ROS levels in metabolic protection. Mitochondrial matrix superoxide can be transported out of the mitochondrial matrix by inner mitochondrial membrane (IMM) anion transporters, where it is converted to H_2_O_2_ by SOD1 in the mitochondrial intermembrane space, from where the H_2_O_2_ can diffuse into the cytoplasm [276]. The deletion of the mitophagy gene Parkin in WAT led to increased mitochondrial superoxide generation that diffused out of the mitochondrial matrix to activate cytoplasmic Nrf2, which induced its target gene NAD(P)H quinone dehydrogenase 1 (NQO1). NQO1, in an enzyme activity-dependent manner, stabilized PGC-1α protein to increase mitochondrial biogenesis, which protected the mice from obesity when they were fed a high-fat diet [277]. Due to the potential presence of the mitochondrial isocitrate-α-ketoglutarate NADPH shuttle in WAT, decreased cytoplasmic [NADPH]/[NADP^+^] can be transmitted to the mitochondrial matrix where it decreases GSR activity to decrease matrix space GSH/GSSG and increase matrix space H_2_O_2_ levels. Therefore, the metabolic health provided by the knockout of either mitochondrial SOD2 or cytoplasmic TKT in WAT may be driven by a similar molecular mechanism that may also be activated by decreased cytoplasmic [NADPH]/[NADP^+^] that occurs during DR. But whether this lowered NADPH level occurs directly after feeding, during the fasting period, or during both (or neither) of these times remains to be established. As WAT cytoplasmic NADPH generation partly relies upon PPP flux, decreased NADPH generation during the DR fasting period, when glucose levels decline slightly, likely drives the metabolic benefits.

## 24. NADPH Inhibits HDAC3, a Class I HDAC, and Class I HDAC Inhibitors Extend Lifespan in Model Organisms

It has been shown that the WAT-specific knockout of the lipogenic genes ACACA or FASN in mice leads to the browning of WAT. In other experiments, the authors showed that the increased acetyl-CoA or malonyl-CoA levels that may occur in WAT when fatty acid synthesis is inhibited do not stimulate the browning of WAT. So, they concluded that decreased levels of free fatty acids or related metabolites signal the browning [278]. However, another possibility is that a moderate increase in [NADPH]/[NADP^+^], which occurs when fatty acid synthesis is inhibited, signals for WAT browning, although, as mentioned earlier, there is other evidence described above that large increases in cytoplasmic NADPH in WAT stimulate lipogenesis and decreased cytoplasmic NADPH stimulates WAT browning. However, consistent with this hypothesis, increased [NADPH]/[NADP^+^] inhibits HDAC3 [279], and HDAC3 inhibition or knockout stimulates the browning of WAT [280,281]. For an overview of HDAC3’s function in the regulation of metabolism, see the following review [282].

There are four class I HDACs in mammals: HDAC1, HDAC2, HDAC3, and HDAC8. NADPH strongly inhibits human HDAC3 [279], while it slightly stimulates the activities of HDAC1 and HDAC2 [283]. NADPH was shown to inhibit HDAC3 by binding the inositol tetraphosphate binding site to prevent the binding of the NCOR1 or NCOR2/SMRT co-repressors needed to activate deacetylase activity [279]. The inhibition or knockdown of HDAC3 activates Nrf2 to protect against oxidative stress [284]. Class I HDAC inhibitors have been shown to extend the lifespan of yeast, *C. elegans*, and *Drosophila* [285]. Under some conditions, HDAC3 represses the browning of WAT, so WAT-specific knockout of HDAC3 in mice on a normal diet led to WAT browning, but WAT browning did not occur when the mice were fed a high-fat diet [280]. The increased mitochondrial gene expression, cytochrome abundance, and UCP1 levels that cause the browning of WAT and increased thermogenesis positively correlate with increased metabolic health and longevity [286]. The expression of mitochondrial-targeted genes causing the browning of WAT could be induced by broad-class I HDAC inhibitors or by HDAC3-specific inhibitors but not by other inhibitors of specific HDACs [281].

In one study, an HDAC3 inhibitor, but not HDAC3 gene knockdown, could induce UCP1 expression as a marker of WAT browning, suggesting that the HDAC3 protein may play a non-enzymatic role in WAT browning under some conditions. However, the addition of isoproterenol, a beta-adrenergic receptor agonist, decreased HDAC3 levels and stimulated UCP1 expression [287], so this non-enzymatic role of HDAC3 may not always be essential or may be accommodated by very low levels of the protein. Others confirmed the ability of an HDAC inhibitor to induce the browning of WAT and showed that HDAC3 inhibition activated the transcriptional regulator of thermogenesis PRDM16 [287], which induces the expression of PGC-1α, PGC-1β, PPAR-γ, and UCP1 [288,289]. The ability of HDAC3 silencing to induce adipocyte browning was shown only to occur in adipocyte precursor cells, not in fully differentiated adipocytes, and the preadipocyte browning was driven by increased levels of PGC-1β but not PGC-1α [242]. Consistent with the ability of class I HDAC inhibition to stimulate WAT browning, it also was shown to inhibit lipogenesis and adipogenesis in 3T3-L1 preadipocytes cultured in differentiation medium, blunting the increased expression of SREBP-1c, PPAR-γ, and C/EPBα [290].

Mesenchymal stem cells (MSCs) are an important source of preadipocytes and adipocytes. Aging MSCs lose their ability to differentiate into the proper cell types, in part through mitochondrial dysfunction, leading to the degradation of the mitochondrial SLC25A1 citrate and isocitrate transporter that decreases cytoplasmic acetyl-CoA levels, lipogenesis, and histone acetylation [291]. Some histone acetylation could potentially be restored by increasing NADPH levels to inhibit HDAC3 and restore MSC regenerative potential to produce functional adipocytes that readily undergo browning to improve lipid catabolism and possibly extend lifespan. Although the histone hypoacetylation that occurs with aging favors MSC differentiation into adipocytes over osteoblasts [292], these aged adipocytes do not respond properly to cues that induce browning, leading to a loss of browned, also called beige, adipocytes in aged individuals [293,294]. In contrast to its function in WAT, hepatic HDAC3 represses lipogenic genes, so liver-specific HDAC3 knockout mice develop fatty liver [295]. Surprisingly, HDAC3 was required for the thermogenic program in BAT [291]. These opposite roles in WAT and BAT may, in part, explain the whitening of BAT that has been shown to occur in DR mice [26]. The DR-induced increase in the expression of BAT ATP synthase inhibitor ATP5IF1 and NCOR1 [7], which functions with HDAC3 to repress the expression of genes involved in mitochondrial oxidative metabolism [296,297], may also play a role.

The similar effects of class I HDAC inhibition and DR in mammals are also consistent with data relating to *Drosophila* that showed that class I HDAC Rpd3 heterozygous flies are long-lived, with a partially overlapping mechanism as that induced by DR [298,299]. A similar molecular mechanism driving the increased replicative longevity was also shown for both glucose-restricted and HDAC Rpd3-deleted yeast, as no additive effect on longevity was observed [300]. Therefore, DR likely increases histone acetylation as an evolutionarily conserved strategy for cytoprotection and longevity. Transcriptional regulators commonly upregulated by class I HDAC inhibitors that are also induced by DR in WAT include NR4A1, FOS [301], and ATF3 [302].

HDACs can remove other histone acyl groups besides the acetyl group, such as the beta-hydroxybutyryl (BHByl) group [303]. Recent data from fasted mice and mice on a ketogenic diet suggest that increased histone beta-hydroxybutyrylation (BHBylation) rather than increased histone acetylation may drive the cytoprotective and longevity-inducing gene expression in the liver, kidney, heart, and muscle [304]. In addition, it was shown that at physiological BHB levels, HDAC1, HDAC2, or HDAC3 can reversibly add or remove BHByl groups to or from histone lysines without the use of a BHByl-CoA intermediate [305]. Together, these studies suggest that the administration of class I HDAC inhibitors to vertebrates might block the protective histone BHBylation that occurs with the DR diet. Longevity-promoting therapies might, therefore, include consuming a general class I HDAC inhibitor, such as butyrate, or an HDAC3-specific inhibitor, before meals before beta-hydroxybutyrate levels start to decline, but not during fasting, when beta-hydroxybutyrate levels are rising to increase histone BHBylation.

## 25. Does a Circadian or Feeding-Induced Increase in WAT [NADPH]/[NADP^+^] Inhibit HDAC3, Leading to the Circadian Increase in Mitochondrial and Lipogenic Gene Expression?

Strikingly, WAT-specific knockout of HDAC3 phenocopied DR by inducing the expression of genes for both fatty acid synthesis and beta-oxidation as well as those for mitochondrial biogenesis, leading to the browning of WAT [280,306]. In mouse liver, high levels of the circadian transcriptional repressor Rev-erbα (NR1D1) recruit HDAC3 to chromatin during the inactive daylight portion of the 24 h cycle, repressing lipogenic gene expression [307] to activate gluconeogenesis [308], even though liver HDAC3 protein levels do not fluctuate in a circadian manner. In addition to low levels of Rev-erbα during the dark phase of the circadian cycle when the mice are actively feeding, the high rate of NADPH synthesis at this time may also increase NADPH levels to inhibit HDAC3, ensuring that HDAC3 does not repress the expression of the lipogenic genes when they are needed during feeding.

HDAC3 inhibition was shown to stimulate mitochondrial oxidative capacity in WAT, the liver, and intestine but was shown to decrease mitochondrial oxidative capacity in skeletal muscle, heart, BAT, and lung [309]. DR-mediated mitochondrial biogenesis appears to be much greater in WAT than in the liver [12]. It is unknown if HDAC3 deacetylates histone lysine residues in a circadian manner in WAT as it does in the liver [310] to stimulate lipogenic gene expression. In fact, the activation of HDAC3 by Zn^2+^ was shown to decrease lipogenesis and increase lipolysis and beta-oxidation in WAT [311]. After a meal, cold-induced mitochondrial biogenesis is inhibited in WAT by glucose-derived acetyl-CoA that is used by p300/CBP to acetylate and stabilize CREBZF, an inhibitor of PGC-1α. During fasting, when blood glucose levels decline, the CREBZF acetylation mark is removed by HDAC3 to activate PGC-1α and mitochondrial biogenesis [312]. These data appear to be inconsistent with the browning of WAT in the WAT-specific HDAC3 knockout mouse [280]. However, HDAC3 inhibition has different effects on terminally differentiated white adipocytes and on white adipocyte precursors [242], and HDAC3 knockout has different metabolic effects in WAT when mice are fed normal or high-fat diets. So, clearly, further studies relating to HDAC3 function in WAT are needed, including studies focusing on the conditional knockout of WAT HDAC3 in mice starting from adulthood fed normal, high-fat, or DR diets. These data lead to many open questions on if and how circadian and feeding and fasting-induced oscillations in [NADPH]/[NADP^+^] regulate lipid metabolism in the liver and WAT. Does [NADPH]/[NADP^+^] peak in WAT in the active phase and bottom out during the inactive phase of the circadian cycle, as might be expected from the circadian activity of SREBP-1c and enzymes that reduce NADP^+^ to NADPH? Does DR amplify the circadian oscillations in [NADPH]/[NADP^+^]? If so, does this lead to greater NADPH-mediated HDAC3 inhibition and the browning of WAT?

Increased mitochondrial biogenesis in several tissues was reported during 30% DR [313], while another group found no evidence for this effect under the same 30% DR protocol [314]. It is possible that mitochondrial biogenesis in the liver and WAT of animals on a DR diet is modulated in response to the meal times to facilitate fatty acid beta-oxidation and fatty acid cycling, so that the data from both groups could be correct, but the mice were sacrificed at different times of the feeding and fasting cycle. A couple of reports suggest that dietary or environmental interventions may amplify circadian oscillations in lipid metabolism. First, a high-fat obesogenic diet synchronized and amplified the circadian oscillations of fatty acid synthesis and beta-oxidation genes in the liver but not WAT [315]. In the second report, chronic cold exposure resulted in high-amplitude circadian oscillations in BAT SREBP1 expression in mice, which activated Rev-erbα and Rev-erbβ (NR1D2) to regulate circadian fluctuations [316]. The knockout of SREBP1 prevented BAT lipogenesis and the maintenance of body temperature. So, both fatty acid synthesis and fatty acid beta-oxidation were shown to be essential for cold-induced thermogenesis in BAT. More research is needed to elucidate the importance of the [NADPH]/[NADP^+^] in circadian WAT lipid metabolism to determine if it plays a key role in WAT browning and DR-mediated longevity.

## 26. Nocturnin, an NADPH Phosphatase Most Highly Expressed in Mouse Liver at Night, Induces PPAR-γ Activity and Lipogenesis

The gene expression of liver 3-hydroxy-3-methylglutaryl-CoA reductase (HMGCR), the rate-limiting enzyme of cholesterol metabolism, a large consumer of NADPH, shows a strong circadian oscillation. For NADPH supply to meet demand, hepatic cytoplasmic NADK shows a circadian gene expression pattern, while mitochondrial NADK2 does not [1,317], although NADK2 expression in WAT is increased by fasting and decreased by a high-fat diet [318]. NADK is also phosphorylated and activated by AKT of the insulin receptor signaling pathway. So, NADK activity further increases following carbohydrate-containing meals when insulin levels are high, providing NADPH for the synthesis of hepatic cholesterol and fatty acids from glucose. Likely due to these circadian oscillations in gene expression, cellular NADPH/NADP^+^ was shown to fluctuate in a circadian manner in zebrafish, which was amplified by hypoxic conditions [319].

Nocturnin, an NADP(H) phosphatase, was shown to be expressed most highly in mouse liver, adipocytes, kidney, and hypothalamus [320]. A cytoplasmic form of the nocturnin gene was shown to be constitutively expressed, while a mitochondrial form was shown to be expressed in a highly circadian manner with peak expression in the liver at night [321]. Nocturnin knockout was shown to protect against high-fat-diet-induced obesity and hepatic steatosis [322]. This is consistent with the role of nocturnin in stimulating adipogenesis by inducing the nuclear translocation of PPAR-γ [320], a master transcriptional regulator of adipogenesis. PPAR-γ also has insulin-sensitizing, anti-inflammatory, and Nrf2-stimulating activities [323,324], and so its expression in mice is positively associated with longevity [325]. Consistent with this, a PPAR-γ agonist has been shown to extend the lifespan of mice [326]. SIRT1 inhibits PPAR-γ expression to stimulate fat mobilization in WAT [327]. DR has been shown to blunt increased hepatic PPAR-γ gene expression with aging [77], perhaps through increased SIRT1 activity. However, the DR diet did not alter hepatic PPAR-γ protein levels, although there was a trend for decreased levels [328]. A high-fat obesogenic diet in mice was shown to decrease hepatic NADP^+^ and NADPH levels without affecting NAD^+^ levels [29], while nicotinamide riboside supplementation partially corrected the deficit [329]. Therefore, therapies that restore liver NADP(H), such as those that increase the circadian oscillations of NADK expression [1], could be useful for the treatment of diabetes.

The knockdown of the *Drosophila* homolog of nocturnin increased mitochondrial fission in Pink1 knockdown cells with decreased mitophagy, suggesting that nocturnin stimulates mitochondrial fusion or inhibits mitochondrial fission [330]. This supports data showing the circadian regulation of mitochondrial dynamics [331]. Mitochondrial fission is associated with increased levels of fatty acyl-CoA [332] and increased rates of fatty acid beta-oxidation in some cells, such as hepatocytes, pancreatic beta-cells, HepG2 hepatocellular carcinoma cells, and B-cell lymphoma cells [333], which could contribute to the protection from obesity observed in nocturnin knockout mice. However, in other hepatocellular carcinoma cells, mitochondrial fission had the opposite effect, increasing fatty acid synthesis by decreasing SIRT1 abundance [334]. In the liver, the genes of the mitochondrial isocitrate-alpha-ketoglutarate NADPH shuttle are highly expressed. By hydrolyzing NADPH to NADH, due to NADPH being present in mitochondria at a much higher concentration than NADP^+^ [71], nocturnin, when induced at night when mice are actively feeding, may prevent mitochondrial [NADPH]/[NADP^+^] from excessively rising and inducing reductive stress. A large pathological increase in mitochondrial [NADPH]/[NADP^+^] would lower [NADP^+^], slowing reactions catalyzed by IDH2, ME3, ALDH1L2, and NNT. The ability of nocturnin to favor mitochondrial fusion over fission may also slow fatty acid beta-oxidation [333], consistent with the ability of nocturnin to stimulate adipogenesis through PPAR-γ [322].

## 27. DR or Increasing the [NADPH]/[NADP^+^] Ratio May Extend Reproductive Longevity and Stimulate the Browning of WAT through an ATF4 and ATF3-Dependent Integrative Stress Response (ISR) Pathway

The knockdown of NADPH phosphatase MESH1 (also called HDDC3) increased NADPH and GSH levels to stimulate glutathione peroxidase 4 (GPX4) activity to protect cells against lipid peroxidation and ferroptosis, while MESH1 overexpression sensitized cells to ferroptosis. NADPH has also been shown to inhibit ferroptosis by activating N-myristoyltransferase 2 (NMT2) to target anti-ferroptotic FSP1 to the plasma membrane [335], where FSP1 oxidizes NADPH to reduce coenzyme Q10 (CoQ10) to prevent phospholipid peroxidation [336]. The ferroptosis-sensitizing activity of MESH1 was antagonized by the increased expression of cytoplasmic NADK but not by mitochondrial NADK2 [337]. The knockdown of MESH1, which increases NADPH levels, induced an ATF4-dependent integrative stress response (ISR) to reestablish cellular NADPH homeostasis [338]. Hepatic ATF4 is activated by many longevity interventions, including DR [73], and contributes to decreased rates of protein synthesis and increased SREBP-1c activation and lipogenesis [339]. The decreased expression of transcription factors ETS1 and ETS2 by DR in WAT [7] may also function along with increased levels of ATF4 to decrease protein synthesis and stimulate longevity [340].

The knockdown of the *C. elegans* homolog of MESH1 called ZK909.3 (*rsh-1*), identified to have a lysosomal localization, did not affect organismal lifespan, although pro-aging TOR kinase was activated [341]. However, ZK909.3 (*rsh-1*) was one of 58 genes that, when knocked down in a global RNAi screen, extended the reproductive longevity of *C. elegans* [342]. DR was shown to extend the reproductive longevity of female mice [343] and fully restored the GSH/GSSG ratio that declined with aging in the testis of male mice [6]. NADPH has recently been shown to be taken up by cells through the P2X purinoceptor 7 receptor (P2X7R) [335]. So, increasing plasma NADPH levels may be a therapeutic option to delay reproductive aging, although many hurdles would need to be overcome to address the short half-life of NADPH in the blood and extracellular fluids.

Decreasing MESH1 to increase cytoplasmic NADPH levels was shown to increase ATF4 levels through a post-transcriptional mechanism, which increased the expression of the ATF4 target gene ATF3 [338]. ATF3 transcriptional activity is involved in the browning of WAT through the inhibition of CHREBP, PPAR-γ, and C/EBPα [344,345]. In rats, WAT ATF3 gene expression decreased with aging, which was restored by DR [7]. Unlike ATF3, studies of ATF4 knockout mice showed that WAT ATF4 stimulated lipogenesis and that BAT ATF4 inhibited UCP1-dependent thermogenesis at ambient temperature [346]. However, another group studying cold-induced thermogenesis showed that ATF4 increased BAT UCP1-independent thermogenesis [347], which may be due to increased futile creatine kinase cycling in BAT [348], as DR increases the expression of creatine kinase B (CKB) in BAT [7]. This is consistent with a previous report that showed futile creatine cycling regulates adipocyte metabolism during intermittent fasting [349]. DR was also shown to decrease the expression of HIF-1α in WAT, which may decrease glycolytic function to increase mitochondrial oxidative metabolism and may contribute to WAT browning [350].

**Figure 3:**
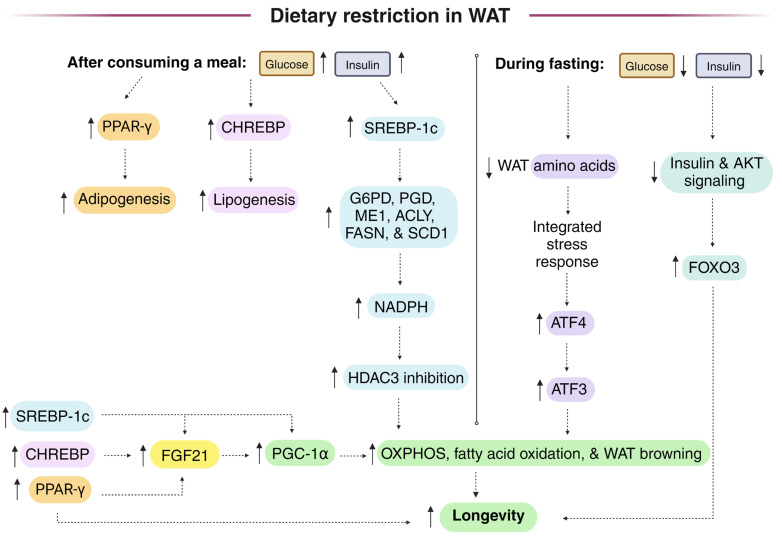
Important transcriptional regulators that play a role in WAT NADPH and lipid metabolism that appear to drive DR-mediated longevity and whether these transcriptional regulators are activated during the feeding or fasting portion of the DR diet. After consuming a meal on the DR diet, the transcriptional regulators SREBP-1c, CHREBP, and PPAR-γ are induced in WAT, which leads to the expression of FGF21, PGC-1α, and lipogenic genes, including cytoplasmic NADPH-generating enzymes. Together, this leads to increased NADPH levels, HDAC3 inhibition, increased mitochondrial biogenesis, the browning of WAT, and fatty acid cycling. For further details, see the following references [145,353]. During the fasting portion of the DR diet, decreased insulin signaling leads to the transcriptional activation of FOXO3, while decreased amino acid levels lead to ISR and ATF4 activation that leads to increased expression of ATF3, which contributes to the browning of WAT, with each contributing to longevity. Dotted arrows represent causation, while solid arrows represent increased or decreased levels and/or activity.

DR or fasting decreases cytoplasmic amino acid levels, activating the ISR, which leads to increased ATF4 translation and increased ATF3 expression [351], likely preventing the aging-induced suppression of the browning of WAT. In rat WAT, DR was also shown to prevent the aging-induced increase in the gene expression of the proinflammatory transcriptional regulators NFκB1, REL (NFκB subunit), and MAFB, while it also prevented the aging-related decrease in the WAT gene expression of the transcriptional regulator C/EBPδ [7]. C/EBPδ has been implicated in mediating some of the changes in WAT gene expression in response to DR [226]. In an scRNA-Seq study, aging decreased the expression of C/EBPβ in 26 of the 42 rat cell types examined, including 18 immune cell types, while expression increased with aging in 4 cell types in WAT. DR restored expression in 22 of these cell types, including 3 of the 4 WAT cell types. DR decreased C/EBPα expression in WAT and BAT [7]. PPAR-γ, C/EBPδ, and another transcriptional regulator, CTCF, have been shown to function together with ATF4 to induce WAT lipogenesis [352]. Figure 3 summarizes the roles of several important transcriptional regulators involved in lipid and NADPH metabolism in WAT and their activation during the feeding or fasting portion of the DR diet and how these regulators may influence DR-mediated longevity.

## 28. DR May Increase the Cytoplasmic [NADPH]/[NADP^+^] in a Limited Number of Tissues in Part through the Activation of Nrf1 and Nrf2, Although Nrf2 Was Not Required for the DR-Mediated Increase in Mouse Longevity

Nrf1 (NFE2L1) and Nrf2 activate gene expression from promoters containing antioxidant response elements (AREs), and DR activates ARE-dependent gene expression [354]. G6PD, PGD, ME1, and IDH1 have AREs in their promoters. For example, the IDH1 promoter has six AREs [355]). In mice, Nrf2 was not required for the lifespan extension that occurs when consuming a DR diet [74]. However, DR-induced longevity is partially abrogated in nematodes deficient in *skn-1a*, the *C. elegans* homolog of Nrf1 [235].

Nrf1 has a membrane-spanning domain and is targeted mostly to the ER membrane. It requires deglycosylation and proteolysis for nuclear translocation and activation. Nrf2 is present in the cytoplasm until activation induces its dissociation from its Keap1 inhibitor, allowing for nuclear translocation. Nrf1 appears to be activated to a small extent constitutively, while Nrf2 is degraded by the proteasome to be present at very low levels under standard conditions until activated by oxidative stress or by small molecules, such as plant-derived polyphenols [356]. This explains the mild phenotype of Nrf2 knockout mice compared to the lethality of the Nrf1 knockout mice [357]. Nrf1 and Nrf2 transcribe sets of overlapping genes. Mammalian IDH1, for example, is induced strongly by Nrf1, but not Nrf2 [358,359], while other NADPH-generating and GSH-synthesizing enzymes appear to be induced by both [359].

It is not yet known if Nrf1 or Nrf2 contribute to the increased expression of NADPH-generating enzymes in WAT that occur when on a DR diet [360], but a scRNA-Seq study of aging and DR in rats, where the rats were placed on the DR diet from 18 to 27 months of age and the data taken from the 27 month DR or ad libitum-fed animals were compared to 5-month-old ad libitum-fed control rats, showed that DR did not increase expression of Nrf2 target genes in the vast majority of the 42 cell types in seven major tissues, although lifespan was extended slightly by the DR diet [7]. However, it is possible that the 18-month middle-aged rats were too old to have Nrf1 or Nrf2 activated. For example, Nrf2 target gene HMOX1 only showed increased expression in two cell types following DR, while aging increased its expression in eight cell types. The Nrf1 target gene of MT1 [361] showed increased expression in 10 cell types in the kidney, liver, skin, and WAT with DR, while it showed increased expression in 19 cell types with aging. NADPH-generating enzymes did not show differential expression with aging or DR, except for IDH1, a Nrf1 target gene, which showed decreased expression in the liver due to DR and decreased expression in the kidney with aging [7]. An RNA-Seq study of mouse liver where DR was initiated at 2 months of age showed similar results, with DR preventing the aging-induced increased expression of the Nrf2 target genes HMOX1, FTH1, NQO1, and GCLC, while DR also decreased MT1 expression, with no change in the expression of SOD2 or IDH1 with aging or DR [77]. Therefore, Nrf2 may only be classically activated in a small number of select cell types during DR to delay increased tumorigenesis with aging. We hypothesize that Nrf1 and Nrf2 may play an important role in the health-inducing protective effects of DR in WAT by stimulating or repressing the activity of other transcriptional regulators rather than by inducing their canonical target genes. Further studies determining the tissue-specific requirements of SKN-1A in *C. elegans* DR-mediated longevity, as well as experiments using Nrf1 WAT-specific knockout mice, are needed to establish a definitive role for Nrf1 in DR-mediated longevity.

Nrf1 may also contribute to increased cytoplasmic NADPH/NADP^+^ by inducing the expression of hepatic pyruvate dehydrogenase kinase 4 (PDK4) [361]. PDK4 phosphorylates and inhibits pyruvate dehydrogenase, shunting the metabolism of pyruvate away from mitochondrial acetyl-CoA to cytoplasmic lactate to increase fatty acid beta-oxidation and decrease NADPH-consuming fatty acid synthesis. Mitochondrial pyruvate is alternatively shunted through pyruvate carboxylase to synthesize oxaloacetate. In the liver, kidney, WAT, pancreatic islets, mammary gland, and adrenal gland increased PDK4 activity can also increase flux through mitochondrial pyruvate carboxylase, where the oxaloacetate synthesized can react with the acetyl-CoA synthesized from fatty acid-beta-oxidation to maintain TCA cycle flux. In the liver and kidney, oxaloacetate can be used for gluconeogenesis. Liver-specific Nrf1 knockout mice show hepatosteatosis, likely in part due to decreased PDK4 levels, activating pyruvate dehydrogenase to stimulate glucose conversion to fatty acids [362]. In mouse liver, DR was shown to prevent the aging-induced increase in PDK4 expression [77]. Nrf1 suppresses WAT adipogenesis via the inhibition of PPAR-γ gene expression [363]. In adipocytes, Nrf1 plays an important role in lipolysis for the mobilization of fatty acids. Therefore, adipocyte-specific Nrf1 knockout leads to adipocyte hypertrophy and inflammation, decreased sWAT mass [364], and a whitening of BAT due to the decreased lipolysis, decreasing levels of fatty acids that fuel thermogenesis [365].

The roles of Nrf2 in the liver and WAT lipid metabolism have been reviewed [366]. Nrf2 is expressed in WAT and has been shown to be required for the browning of WAT [367,368]. Complexly, a mutation hyperactivating Nrf2 showed atrophied eWAT [369]. Unexpectedly, Nrf2-deficient mice showed increased glucose tolerance when fed a high-fat diet, which was shown to be due to the upregulation of SIRT1 in eWAT, leading to increased lipolysis and fatty acid beta-oxidation [370]. In adipocytes, Nrf2 binds the promoter and induces the expression of SREBP-1c, inducing lipogenesis [371]. So, Nrf2-deficient mice show decreased adipocyte mass and size [372]. Tissue-specific knockout of Nrf2 showed that Nrf2-specific deficiency in WAT, but not in liver, potentiated the metabolic dysfunction caused by a high-fat diet [373].

When Nrf2 knockout mice were crossed onto a C57BL/6J background and were fed ad libitum, they showed decreased weight and increased heat production compared to control mice [360]. This could potentially be due to increased fatty acid cycling in eWAT, as described above, but Nrf2 knockout mice also show increased WAT UCP1 levels that may also be responsible for these phenotypes [374]. The Nrf2-deficient mice showed a decreased mean lifespan only during ad libitum fed conditions, as the lifespan was extended by DR, similar to the control mice. On this genetic background, the mice showed a similar increase in weight and decrease in lifespan when on a high-fat diet as the control mice. The Nrf2 knockout mice showed similar, but not identical, changes in daytime and nighttime physical activity and respiratory exchange ratio, a measure of carbohydrate or lipid oxidation, as control mice on a DR or high-fat diet, suggesting that the lack of Nrf2 does not greatly alter DR-induced fatty acid cycling in the mice, consistent with the extended longevity of the Nrf2 knockout mice on the DR diet that likely requires fatty acid cycling [360], as SREBP-1c knockout mice that cannot undergo DR-induced fatty acid cycling did not show extended longevity on the DR diet [12].

Keap1 knockout mice with constitutive Nrf2 activation die by postnatal day 21 due to feeding problems [375]. Studies were performed using Keap1 knockdown mice to determine the role of Nrf2 activation on the metabolic response to fasting [376]. Constitutive Nrf2 activation led to decreased fasting-induced hepatosteatosis compared to WT animals as a result of the decreased expression of lipogenic genes and increased AMPK activation [377]. In WAT, Nrf2 activation did not alter lipolysis but led to the decreased expression of fatty acid transporters, leading to the accumulation of free fatty acids, while the animals showed increased glucose tolerance. Mice possessing two Keap1 hypomorphic alleles to activate Nrf2 placed on a high-fat diet were protected from metabolic dysfunction and showed decreased hepatic lipogenesis and gluconeogenesis [378]. Studies are needed to determine the effects of the DR diet on mice with reduced Keap1 activity to determine the effects on longevity and WAT NADPH and lipid metabolism.

## 29. DR Affects mRNA Splicing That May Affect NADPH Metabolism in Liver and WAT

The *Drosophila* splicing factor 9G8 was shown to be essential for maintaining the expression of the four major genes involved in cytoplasmic NADPH generation, including G6PD, PGD, IDH, and Men, in the fat body [379]. Proteomics of the liver of non-human primates on a DR diet showed a decreased abundance of the human homolog of 9G8 called SRSF7. Therefore, decreased SRSF7 levels may decrease NADPH generation during the fasting times of the DR diet, when fatty acids are not actively being synthesized. SRSF7 is important in young tissues to support anabolism and growth, which require high levels of NADPH. The abundance of hepatic SRSF7 decreased with aging. In rats, NADPH levels were shown to be highest during embryonic and fetal development and continued to decline until 8 days after birth [380]. The depletion of SRSF7 was shown to increase the levels of pyruvate dehydrogenase kinase 1 (PDK1) and pyruvate dehydrogenase kinase 2 (PDK2) [381], which function similarly to PDK4 described above, to inhibit pyruvate dehydrogenase activity. SRSF7 also alters the splicing of proteins involved in mitochondrial fission in the liver [381]. SRSF7 and SRSF2 were shown to be required for mTOR activation [382], while mitochondrial dysfunction increased the expression of SRSF2 to induce cytoprotective mitophagy [383,384]. There was an aging-related decrease in SRSF2 expression in rat WAT, which was further decreased by DR [7]. DR increased the hepatic abundance of several other splicing factors, such as SRSF1 [129], which plays an important role in the splicing of ETC complex I subunit NDUFS3, which is involved in the browning of WAT [385]. SRSF1 deficiency in mouse liver resulted in hepatosteatosis [386], likely due to increased lipogenesis, as mice that were heterozygous for splicing factor SRSF10 were shown to have increased expression of hepatic SREBP-1c and FASN that led to increased lipogenesis and fatty liver [387].

## 30. Mitochondrial SIRT3 Is Induced by DR to Activate IDH2 to Increase Mitochondrial NADPH, but SIRT3 Knockout Mice Maintain Robust Longevity on the DR Diet

Hepatic mitochondrial SIRT3 is activated during DR and deacetylates mouse mitochondrial TCA cycle enzyme IDH2 at K413 [69], increasing enzyme activity 44-fold to increase the reduction of mitochondrial NADP^+^ to NADPH [388]. This lysine residue is also conserved in cytoplasmic IDH1. Unexpectedly, SIRT3 knockout mice on a DR diet lived longer than control mice on a DR diet, although mitochondrial oxygen consumption was decreased [389]. Therefore, a DR-mediated increase in mitochondrial [NADPH]/[NADP^+^] may not be required for the longevity benefits of DR, although SIRT3 is required for DR to delay aging-induced hearing loss [69]. In contrast, SIRT3 knockout mice fed ad libitum on a standard chow diet showed a slightly decreased lifespan [390]. However, it has not definitively been shown that SIRT3 knockout prevents DR from increasing the mitochondrial [NADPH]/[NADP^+^]. This is still unknown because during DR or fasting, there is also increased hepatic expression of mitochondrial NAD^+^ transporter SLC25A51 [391], and as mentioned earlier, fasting increases the WAT and hepatic expression of NADK2 [318]. In addition, human mitochondrial NAD^+^ kinase NADK2 has been shown to be acetylated at K76 and K304, leading to the inhibition of NADK2 activity [392]. It is not yet known if these NADK2 lysine residues are acylated in vivo or deacylated by mitochondrial SIRT3 or SIRT5 during DR to activate NADK2. If NADK2 is deacylated by SIRT5, this could lead to a DR-mediated increase in mitochondrial [NADPH]/[NADP^+^] in the SIRT3 knockout mice, as an increase in NADK activity has been shown to increase NADPH/NADP^+^ [393].

Mouse liver NADK2 K360, which has an arginine residue in this position in the orthologous human sequence, showed increased acetylation in SIRT3 knockout mice [394]. The K360 residue is present in the NADK2 dimerization region needed for enzymatic activity [392]. So, studies should be performed to determine if the acetylation of mouse NADK2 K360 affects dimerization and enzyme activity and to determine if SIRT3-mediated deacetylation of this site during DR regulates NADK2 activity. The NADK2 K360 lysine-to-arginine switch in humans compared to mice might explain differences in mitochondrial NADPH metabolism between the two species. Future studies focusing on NADK2 function should not use C57BL/6J mice, as this strain lost the normal stop codon for the NADK2 gene [395], in addition to the more well-known loss of the NNT gene in C57BL/6J mice, affecting mitochondrial NADPH metabolism [396].

## 31. Post-Translational Mechanisms through Which DR May Increase the Cytoplasmic [NADPH]/[NADP^+^]

Although cytoplasmic [NAD^+^]/[NADH] is reduced in the liver of mice on a DR diet [5], liver SIRT1 levels have been found to either increase [397] or decrease [70] in DR mice, likely depending upon circadian rhythms or the feeding schedule. The NAD^+^/NADH in WAT increases during DR [70], accompanied by the increased expression of SIRT1 and SIRT2 [398], which are also activated by the increased NAD^+^ level. G6PD has two binding sites for NADP^+^, a catalytic site and a structural site. NADP^+^ binding to the structural site is required for G6PD dimerization and enzyme activity, while tetramerization likely increases enzyme activity further [399]. Human G6PD is acetylated on several lysine residues, including K403, which blocks the binding of the structural NADP^+^ and prevents enzymatic activity [399,400]. Both SIRT1 and SIRT2 have been shown to deacetylate K403 to activate G6PD [400], while KAT9 [401] and CREB-binding protein (CBP) acetylate K403 and inactivate the enzyme [400]. For a review of the many G6PD post-translational modifications, the following reference is recommended [118].

Mitochondrial protein acetylation is high in the liver and kidney due to their high mitochondrial acetyl-CoA levels [394,402]. In fact, 65% of all mouse liver mitochondrial proteins were shown to be acetylated. In the liver, 69% of the 1757 quantifiable protein lysine acetylation sites were found on mitochondrial proteins [402]. Using twofold change as a cutoff, DR was shown to increase the acetylation of 135 mitochondrial acetylation sites, while a similar number of acetylation sites showed decreased acetylation. Cytoplasmic acetylation sites were much less affected, which is likely due to the reduced hepatic cytoplasmic [NAD^+^]/[NADH] during DR, which tends to decrease hepatic cytoplasmic sirtuin activity. DR has been shown to decrease liver acetyl-CoA levels fivefold, mostly reflecting cytoplasmic changes [402], but fasting increased mitochondrial acetyl-CoA levels threefold [66]. However, this increase in acetyl-CoA that stimulates acetylation is compensated for by the increased levels of mitochondrial SIRT3 that stimulate mitochondrial protein deacetylation during DR [402,403]. SIRT3 knockout caused roughly four times as many changes in the protein acetylation site status as DR [402]. Therefore, changes in protein acetylation status completely remodel the mitochondrial metabolism of animals on a DR diet, greatly influencing redox metabolism, while the cytoplasmic redox metabolism is more indirectly affected through the activity of redox shuttle systems that relay signals to the cytoplasm, with a few exceptions, as described below.

SIRT2 deacetylates and activates IDH1. Human IDH1 activity was shown to be inhibited 5 to 10-fold by the acetylation of K81, K224, or K321 [404]. In cultured cells, IDH1 was shown to be deacetylated at K224 by SIRT2 [83] or by SIRT1 [84,85]. In mouse liver, IDH1 was shown to be acetylated on nine different lysine residues, while DR led to the deacetylation of IDH1 on K58, K224, and K243, with the K224 deacetylation mediating activation during DR [402]. DR also led to the deacetylation of cytoplasmic NADPH-generating folate cycle enzyme ALDH1L1 on K660 in mouse liver [402]. However, the effects of this modification are not yet known.

Lysine BHBylation is another post-translational modification that may potentially activate enzymes that reduce NADP^+^ to NADPH during DR, as plasma beta-hydroxybutyrate levels can increase 10- to 100-fold during fasting and DR [405,406]. IDH2, GLUD1, and NADK2, as well as several enzymes of the folate and methionine cycles, such as SHMT1 and AHCY, were shown to be beta-hydroxybutyrylated (BHBylated) in the liver of fasted mice [407]. AHCY BHBylation inhibited enzyme activity [407], but DR prevented the aging-induced decrease in gene expression of AHCY and another folate cycle enzyme, glycine N-methyltransferase (GNMT) [77]. Like acetylation, BHBylation levels are highest in the liver and kidney during fasting. Following fasting in the liver, ME1 and ME3 have been shown to be BHBylated at an allosteric activator site [408]. BHBylation was also detected at sites in the MAS enzymes mitochondrial malate dehydrogenase (MDH2) and mitochondrial aspartate aminotransferase (GOT2) that are predicted to affect enzymatic function [408].

## 32. Conclusions

Blunted circadian oscillations in WAT cytoplasmic [NADPH]/[NADP^+^] or blunted changes to this ratio in response to feeding and fasting cycles could be major driving forces for the metabolic dysfunction that occurs with organismal aging. It is hypothesized that DR restores youthful daily oscillations in the [NADPH]/[NADP^+^] in WAT, which are key to the health and longevity benefits of the DR diet. The lack of facile and reliable methods to measure the cytoplasmic [NADPH]/[NADP^+^] in tissues, especially in the longevity-regulating tissue WAT, has limited progress in the field. The use of newly developed fluorescent biosensor technology for monitoring [NADPH]/[NADP^+^] [409] in animal models in vivo, in particular using transparent fish aging models, may soon be able to shine a light on the importance of changes in the cytoplasmic [NADPH]/[NADP^+^] in aging and anti-aging DR. Alternatively, the NADPH-oxidizing enzyme triphosphopyridine nucleotide oxidase (TPNOX) could be expressed in vertebrate models in a tissue-specific manner to selectively lower cytoplasmic [NADPH]/[NADP^+^] [410] to determine the effects on the metabolic and longevity benefits of DR.

Five non-mutually exclusive mechanisms are suggested through which SREBP-1c activation in WAT during DR may lead to lifespan extension. (1) The first mechanism is by diurnally increasing NADPH synthesis and cytoplasmic [NADPH]/[NADP^+^] during feeding, leading to HDAC3 inhibition and ISR activation, increasing the expression of lipogenic and longevity-promoting genes, while this is followed by fasting and likely a diurnal decrease in cytoplasmic [NADPH]/[NADP^+^] when WAT lipolysis is activated. (2) The second mechanism involves WAT SREBP-1c increasing lipid synthesis during DR to a greater extent than NADPH synthesis to decrease WAT [NADPH]/[NADP^+^], which leads to increased H_2_O_2_ that inhibits glycolysis and stimulates fatty acid beta-oxidation, oxidative phosphorylation, and the browning of WAT. (3) The third mechanism involves fatty acid cycling and the replacement of highly unsaturated membrane dietary fatty acids with more saturated WAT-synthesized fatty acids, which leads to DR-mediated protection from ferroptosis. (4) The fourth mechanism involves the increased synthesis of WAT MUFAs that are released to peripheral tissues, where they induce lipid droplet synthesis and activation of prolongevity transcriptional regulator Nrf1. (5) The fifth mechanism involves fatty acid cycling between WAT and the liver and the activation of the NADPH-FADH_2_ axis that decreases hepatic mitochondrial superoxide production. Through one or more of these mechanisms, DR and its induction of SREBP-1c in WAT likely lead to large changes in WAT NADPH and lipid metabolism, driving lifespan extension.

## Figures and Tables

**Table 1:** Measurements of the redox state of total liver pyridine nucleotides frequently do not yield values indicative of the redox state of the free cytoplasmic nucleotides that regulate metabolism.

	Aging	DR	Fasting
Liver free cytoplasmic [NADPH]/[NADP^+^]	No change [5]	Increased [5]	Increased [30,66]
Liver total NADPH/NADP^+^	Decreased [68]	Increased [69]	No change [30,66]
Liver free cytoplasmic [NAD^+^]/[NADH]	No change [5]	Decreased [5]	Decreased [30,66]
Liver total NAD^+^/NADH	Increased [68]	Decreased [70]	No change [30,66]

**Table 2:** Major cytoplasmic and mitochondrial enzymes that reduce NADP^+^ to NADPH.

Enzyme	Pathway	Localization	Information
G6PD	PPP	cytoplasmic	Largely inhibited by NADPH in vivo; deacetylated at K403 by SIRT1 or SIRT2 activates G6PD
PGD	PPP	cytoplasmic	Activated by ME1; inhibited by malate
ME1	Citrate-pyruvate shuttle	cytoplasmic	High expression in lipogenic tissues; absent from neurons; activated by beta-hydroxybutyrylation
IDH1	Isocitrate-α-ketoglutarate shuttle	cytoplasmic	Major NADP^+^-reducing enzyme in heart and skeletal muscle; absent from normal astrocytes, but induced by cell division in cancer cells
ALDH1L1	Folate cycle	cytoplasmic	Major cytoplasmic NADP^+^-reducing enzyme in liver
MTHFD1	Folate cycle	cytoplasmic	Oxidizes NADPH in most tissues, but reduces NADP^+^ in liver where folate cycle flux is reversed
IDH2	Isocitrate-α-ketoglutarate shuttle	mitochondrial	Major mitochondrial NADPH generator Deacetylation by SIRT3 activates IDH2
ME3		mitochondrial	Activated by beta-hydroxybutyrylation
NNT		mitochondrial	NADPH generation driven by IMM proton gradient Mutated in C57BL/6J, but not C57BL/6N mice
ALDH1L2	Folate cycle	mitochondrial	Deacetylation by SIRT3 activates ALDH1L2
MTHFD2	Folate cycle	mitochondrial	NAD^+^ and NADP^+^ compete for the active site
MTHFD2L			
GLUD1	Glutaminolysis	mitochondrial	NAD^+^ and NADP^+^ compete for the active site

## Supplementary Materials

The following supporting information can be downloaded at: https://www.mdpi.com/article/10.3390/antiox13070820/s1, Table S1: Changes in gene expression and circadian cycling of gene expression in young and aged liver for mice on ad libitum and 5 different isocaloric DR diets, where the feeding times were altered over the circadian cycle, for over 300 select genes involved in NADPH and lipid metabolism and that could potentially play a role in DR-mediated longevity.

## Abbreviations

ACACA	acetyl-CoA carboxylase-alpha
ACLY	ATP-citrate lyase
ACO1	aconitase 1—cytoplasmic
ACO2	aconitase 2—mitochondrial
AHCY	adenosylhomocysteinase
ALDH1L1	cytosolic 10-formyltetrahydrofolate dehydrogenase
ALDH1L2	mitochondrial 10-formyltetrahydrofolate dehydrogenase
AMPK	AMP-activated protein kinase
ATIC	5-aminoimidazole-4-carboxamide ribonucleotide formyltransferase/IMP cyclohydrolase
ATF3	activating transcription factor 3
ATF4	activating transcription factor 4
AREs	antioxidant response elements
BAT	brown adipose tissue
BCAA	branched chain amino acid
BHByl	beta-hydroxybutyryl
BHBylated	beta-hydroxybutyrlated
BHBylation	beta-hydroxybutyrylation
CBP	CREB binding protein
CBS	cystathionine beta-synthase
C/EBPα	CCAAT enhancer-binding protein alpha
C/EBPδ	CCAAT enhancer-binding protein delta
CHREBP	carbohydrate response element-binding protein
CoA	coenzyme A
CoQ10	coenzyme Q10
CO_2_	carbon dioxide
CS	citrate synthase
CTH	cystathionine gamma-lyase
DHF	dihydrofolate
DHFR	dihydrofolate reductase
DOT1L	DOT1-like histone lysine methyltransferase
dTMP	deoxythymidine monophosphate
dUMP	deoxyuridine monophosphate
DUOX2	dual oxidase 2
DR	dietary restriction
ER	endoplasmic reticulum
ERAD	ER-associated degradation
ETC	electron transport chain
eWAT	epididymal white adipose tissue
FAD	flavin adenine dinucleotide—oxidized form
FADH_2_	flavin adenine dinucleotide—reduced form
FASN	fatty acid synthase
FGF21	fibroblast growth factor 21
10-formylTHF	10-formyltetrahydrofolate
FOS	Fos proto-oncogene, AP-1 transcription factor subunit
FOXA1	forkhead box A1
FOXA2	forkhead box A2
FOXA3	forkhead box A3
FOXO1	forkhead box O1
FOXO3	forkhead box O3
FOXO4	forkhead box O4
FOXO6	forkhead box O6
FSP1	ferroptosis suppressor protein 1 encoded by AIFM2 gene
FTO	fat mass and obesity-associated
GAPDH	glyceraldehyde-3-phosphate dehydrogenase
GART	phosphoribosylglycinamide formyltransferase, phosphoribosylglycinamide synthetase, and phosphoribosylaminoimidazole synthetase
GCLC	glutamate-cysteine ligase catalytic subunit
GCS	glycine cleavage system
GLUD1	glutamate dehydrogenase 1
GNMT	glycine N-methyltransferase
GOT2	glutamate-oxaloacetate transaminase 2—mitochondrial
GPX4	glutathione peroxidase 4
GSH	glutathione
GSR	glutathione disulfide reductase
GSS	glutathione synthetase
GSSG	glutathione disulfide
GYG1	glycogenin 1
HDAC1	histone deacetylase 1
HDAC2	histone deacetylase 2
HDAC3	histone deacetylase 3
HDAC6	histone deacetylase 6
HDAC8	histone deacetylase 8
HMGCR	3-hydroxy-3-methylglutaryl-CoA reductase
HSCARG	NADPH sensor encoded by the NMRAL1 gene
H_2_O_2_	hydrogen peroxide
IDH1	isocitrate dehydrogenase 1
IDH2	isocitrate dehydrogenase 2
IMM	inner mitochondrial membrane
ISR	integrated stress response
JAZF1	JAZF zinc finger 1
JUNB	JUNB proto-oncogene, AP-1 transcription factor subunit
KAT9	lysine acetyltransferase 9 encoded by the ELP3 gene
LDH	lactate dehydrogenase
LXRα	liver X receptor-alpha—encoded by NR1H3 gene
LXRβ	liver X receptor-beta—encoded by NR1H2 gene
MAFB	MAF bZIP transcription factor B
MAS	malate-aspartate shuttle
MAT1A	methionine adenosyltransferase 1A
MAT2A	methionine adenosyltransferase 2A
MAX	myc-associated factor x
MDH1	malate dehydrogenase 1—cytoplasmic
MDH2	malate dehydrogenase 2—mitochondrial
MESH1	metazoan SpoT homolog 1
5, 10-methenylTHF	5, 10 methenyltetrahydrofolate
5, 10-meTHF	5, 10-methylenetetrahydrofolate
5-meTHF	5-methyltetrahydrofolate
ME1	malic enzyme 1
ME3	malic enzyme 3
MLX	max dimerization protein
MPC	mitochondrial pyruvate carrier
MPTP	1-methyl-4-phenyl-1,2,3,6-tetrahydropyridine
MSCs	mesenchymal stem cells
MTHFD1	methylene tetrahydrofolate dehydrogenase 1
MTHFD2	methylene tetrahydrofolate dehydrogenase 2
MTHFD2L	methylene tetrahydrofolate dehydrogenase 2-like
MTHFR	methylenetetrahydrofolate reductase
mTORC1	mechanistic target of rapamycin complex 1
MTR	5-methyltetrahydrofolate-homocysteine methyltransferase (methionine synthase)
MUFAs	monounsaturated fatty acids
m^6^A	N^6^-methyladenosine
NAD^+^	nicotinamide adenine dinucleotide—oxidized form
NADH	nicotinamide adenine dinucleotide—reduced form
NADK	NAD^+^ kinase—cytoplasmic
NADK2	NAD^+^ kinase 2—mitochondrial
NADP^+^	nicotinamide adenine dinucleotide phosphate– oxidized form
NADPH	nicotinamide adenine dinucleotide phosphate– reduced form
NCOR1	nuclear receptor corepressor 1
NCOR2	nuclear receptor corepressor 2
NDUFA5	NADH:ubiquinone oxidoreductase subunit A5
NDUFS1	NADH:ubiquinone oxidoreductase subunit S1
NDUFS2	NADH:ubiquinone oxidoreductase subunit S2
NDUFS3	NADH:ubiquinone oxidoreductase subunit S3
NDUFV2	NADH:ubiquinone oxidoreductase subunit V2
NFκB1	nuclear factor NF-kappa-B p50 DNA-binding subunit
NH_3_	ammonia
NMDMC	methylenetetrahydrofolate dehydrogenase 2 (MTHFD2) mito. (*Drosophila melanogaster*)
NMT2	N-myristoyltransferase 2
NNT	nicotinamide nucleotide transhydrogenase
NPCs	neural progenitor cells
Nrf1	NFE2L1
Nrf2	NFE2L2
PDHB	pyruvate dehydrogenase B subunit
PDK1	pyruvate dehydrogenase kinase 1
PDK4	pyruvate dehydrogenase kinase 4
PGC-1α	peroxisome proliferator-activated receptor gamma coactivator-1α
PGC-1β	peroxisome proliferator-activated receptor gamma coactivator-1β
PHGDH	3-phosphoglycerate dehydrogenase
P_i_	inorganic phosphate
Pink1	PTEN-induced kinase 1
PPAR-γ	peroxisome proliferator-activated receptor gamma
PPP	pentose phosphate pathway
PRDM16	PR/SET domain 16
PRDX5	peroxiredoxin 5
PSAT1	phosphoserine aminotransferase 1
PSPH	phosphoserine phosphatase
PUFAs	polyunsaturated fatty acids
P2X7R	P2X purinoceptor 7 receptor
RBC	red blood cell
redox	oxidation–reduction
REL	REL proto-oncogene, NF-κB subunit
Rev-erbα	nuclear receptor Rev-erbA-alpha encoded by the NR1D1 gene
Rev-erbβ	nuclear receptor Rev-erbA-beta encoded by the NR1D2 gene
ROS	reactive oxygen species
rWAT	retroperitoneal white adipose tissue
SCAP	SREBP cleavage-activating protein
SCD1	stearoyl-CoA desaturase 1
SDS	serine dehydratase
SHMT1	serine hydroxymethyltransferase 1—cytoplasmic
SHMT2	serine hydroxymethyltransferase 2—mitochondrial
SIRT1	sirtuin 1
SIRT2	sirtuin 2
SIRT3	sirtuin 3
SLC25A1	mitochondrial citrate-isocitrate carrier
SLC25A11	mitochondrial 2-oxoglutarate/malate carrier
SLC25A12	mitochondrial aspartate-glutamate carrier Aralar
SLC25A13	mitochondrial aspartate-glutamate carrier Citrin
SLC25A32	mitochondrial folate and flavin adenine dinucleotide (FAD) carrier
SLC25A51	mitochondrial NAD^+^ carrier
SOD2	superoxide dismutase 2
SREBP1	sterol regulatory element-binding protein 1
SREBP-1c	sterol regulatory element-binding protein-1c
SREBP2	sterol regulatory element-binding protein 2
SRSF1	serine and arginine-rich splicing factor 1
SRSF2	serine and arginine-rich splicing factor 2
SRSF7	serine and arginine-rich splicing factor 7
SRSF10	serine and arginine-rich splicing factor 10
sWAT	subcutaneous white adipose tissue
TALD	transaldolase
TFEB	transcription factor EB
THF	tetrahydrofolate
TPNOX	triphosphopyridine nucleotide oxidase
TKT	transketolase
TXNRD2	thioredoxin reductase 2—mitochondrial
TYMS	thymidylate synthase
UCP1	uncoupling protein 1
UCP2	uncoupling protein 2
UPR^mt^	mitochondrial unfolded protein response
WAT	white adipose tissue
XBP1	x-box binding protein 1
